# Effects and Mechanisms of Action of Preussin, a Marine Fungal Metabolite, against the Triple-Negative Breast Cancer Cell Line, MDA-MB-231, in 2D and 3D Cultures

**DOI:** 10.3390/md21030166

**Published:** 2023-03-04

**Authors:** Rosária Seabra, Fernanda Malhão, Alexandra Correia, Carla Costa, Anake Kijjoa, Eduardo Rocha

**Affiliations:** 1 ICBAS—Instituto de Ciências Biomédicas de Abel Salazar, Universidade do Porto, Rua de Jorge Viterbo Ferreira, 228, 4050-313 Porto, Portugal; 2 CIIMAR—Centro Interdisciplinar de Investigação Marinha e Ambiental, Universidade do Porto, Terminal de Cruzeiros do Porto de Leixões, Av. General Norton de Matos s/n, 4450-208 Ma-tosinhos, Portugal; 3 I3S—Instituto de Investigação e Inovação em Saúde, Universidade do Porto, Rua Alfredo Allen, 4200-135 Porto, Portugal; 4 Department of Environmental Health, INSA—National Institute of Health Dr. Ricardo Jorge, R. de Alexandre Herculano 321, 4000-053 Porto, Portugal; 5 ITR—Laboratório para a Investigação Integrativa e Translacional em Saúde Populacional, EPIUnit—Instituto de Saúde Pública da Universidade do Porto, Rua das Taipas 135, 4050-600 Porto, Portugal

**Keywords:** 3D cell culture, preussin, cytotoxicity, cancer cells death, triple-negative breast cancer

## Abstract

Triple-negative breast cancer (TNBC) represents an aggressive subtype of breast cancer (BC) with a typically poorer prognosis than other subtypes of BC and limited therapeutic options. Therefore, new drugs would be particularly welcome to help treat TNBC. Preussin, isolated from the marine sponge-associated fungus, *Aspergillus candidus*, has shown the potential to reduce cell viability and proliferation as well as to induce cell death and cell cycle arrest in 2D cell culture models. However, studies that better mimic the tumors in vivo, such as 3D cell cultures, are needed. Here, we studied the effects of preussin in the MDA-MB-231 cell line, comparing 2D and 3D cell cultures, using ultrastructural analysis and the MTT, BrdU, annexin V-PI, comet (alkaline and FPG modified versions), and wound healing assays. Preussin was found to decrease cell viability, both in 2D and 3D cell cultures, in a dose-dependent manner, impair cell proliferation, and induce cell death, therefore excluding the hypothesis of genotoxic properties. The cellular impacts were reflected by ultrastructural alterations in both cell culture models. Preussin also significantly inhibited the migration of MDA-MB-231 cells. The new data expanded the knowledge on preussin actions while supporting other studies, highlighting its potential as a molecule or scaffold for the development of new anticancer drugs against TNBC.

## 1. Introduction

Triple-negative breast cancer (TNBC) has a highly aggressive nature and poor prognosis. Contrary to other breast cancer (BC) subtypes, TNBC is characterized by the lack of expression of estrogen receptor (ER), progesterone receptor (PGR), and human epidermal growth factor receptor 2 (HER2) [1,2]. Due to the lack of expression of the molecular targets ER, PGR, and HER2, TNBC has limited therapeutic options, with adjuvant chemotherapy (with regimens of anthracyclines and taxanes used preferably sequentially) as the main recommended treatment option for early, locally advanced, and metastatic BC cases [1,2,3].

Due to TNBC characteristics, chemotherapy-associated toxicity, the development of drug resistance, and early onset of metastasis, the search for compounds aiming at different therapeutic targets or potentiating the existing established drugs, with minimal or at least decreased toxicity towards normal cells, has become a priority to overcome the poor prognosis associated with this disease [4,5].

Since biodiversity can be translated into chemodiversity, Nature represents the most important and vast source of new bioactive molecules [6,7,8]. In their recent review article, Atanasov et al. [9] stated that “Natural products (NPs) are structurally ‘optimized’ by evolution to serve particular biological functions”. The scope of Nature as a source of novel chemical entities and scaffolds has expanded even more with the search for new molecules from the marine environment [8,10]. In the marine environment, marine-derived fungi can be isolated from water and sediments, as well as from macroorganisms with which they are associated, such as corals, sea cucumber, snails, and sponges as well as algae and mangrove plants [11,12]. Marine sponges are an important source of bioactive compounds, some of which have served as models for anticancer drugs while others showed great potential as lead compounds for anticancer drug discovery [13,14,15]. In the marine sponges’ filtration process, some microorganisms may be phagocytized by the sponges, but others remain in the sponges’ mesohyl (the inner sponge cell lining). Some of these microorganisms may then develop symbiotic interactions with the sponges, becoming involved in their physiological processes, including nutrition, skeleton stabilization, metabolite production, and chemical defense [13,16]. Thus, some of the bioactive substances discovered from the marine sponges, mostly secondary metabolites, are biosynthesized by marine sponge-associated microorganisms rather than by the sponges themselves [13].

Although the density of microorganisms in marine sponges vary greatly, they may account for up to 40% of the sponge volume [13,16]. The diverse conditions (including the symbiotic interactions with other organisms) to which the marine-derived fungi are subjected lead to the development of unique metabolic pathways that may, in turn, lead to the production of a wide variety of natural products with potential pharmacological properties, including anticancer activity [12,13,17,18,19]. Therefore, marine-derived fungi represent an exciting source of bioactive compounds with unique structural features with interesting pharmacological properties, including anticancer properties that deserve further investigation.

**Figure 1:**
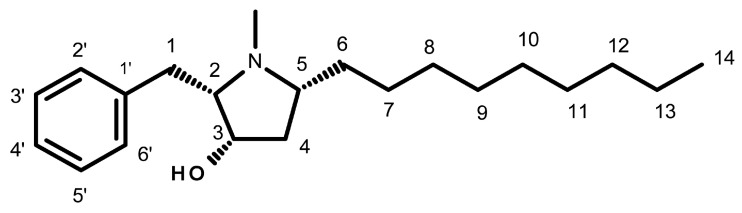
Chemical structure of preussin.

Preussin is a hydroxypyrrolidine alkaloid (Figure 1) that was initially isolated from the fermentation broth extracts of *Aspergillus ochraceus* [20,21], and later from *Preussia* sp. [22]. More recently, preussin was also obtained from the culture extract of *A. candidus* KUFA 0062, isolated from a marine sponge, *Epipolasis* sp., which was collected from the Similan Islands National Park’s coral reef, in Phang-Nga province, Southern Thailand [23]. Given the importance of preussin, various authors have described different methods for its synthesis [21,24,25,26,27,28,29,30,31,32,33,34,35,36,37].

Several preussin analogs have been identified and isolated from natural sources. One is (+)-preussin (Figure 1), which is commonly known as preussin. Others are preussin B, isolated from extracts of the fungus *Simplicillium lanosoniveum* [38], preussin C, isolated from the marine sponge-associated fungus *A. candidus* [23] and *A. flocculosus* [39], and preussins D-K, obtained from the fermentation broths of the marine sponge-associated fungus *A. flocculosus* [23,38,39]

Although there is not much literature on preussin and its isomers, (+)-preussin is the most described and seems to have a broader spectrum of biological activities. Preussin has shown antifungal activities against both filamentous fungi and yeast [20,22,40]. It also exhibited bacteriostatic and bactericidal properties against Gram-positive bacteria, as well as an ability to inhibit methicillin- and vancomycin-resistant bacterial strains [23], in addition to antiviral properties [41].

Preussin has also been shown to display cytotoxic activities [23,27,42,43]. Achenbach et al. [42] showed that synthetic preussin decreased cell survival in several human cancer cell lines, with half-maximal inhibitory concentrations (IC_50_) ranging from 1.2 to 4.5 µM. Buttachon et al. [23] found that preussin, isolated from the marine sponge-associated fungus, *A. candidus*, decreased cell viability, with variable IC_50_ values, ranging from 12.3 µM to 74.1 µM, while Malhão et al. showed that the same compound (at 50 or 100 µM) decreased cell viability below 50% in various human breast cancer cell lines [43].

Preussin also induced apoptosis in promyelocytic leukemia HL-60 cell line [42] and increased the expression of caspase-3 in various breast cancer cell lines [43]. In these cell lines, preussin decreased cell proliferation in monolayer (at 25 µM) and 3D cell cultures (at 50 µM), with the highly aggressive MDA-MB-231 cell line showing higher resistance [43]. Preussin also promoted cell cycle arrest in cancerous and normal cell lines [42]. Finally, preussin was shown to be a DNA-damaging agent in *Saccharomyces cerevisiae* [44].

The diverse biological activities of preussin have been attributed to some of its molecular characteristics. Buttachon et al. emphasized the importance of the *N*-methyl group on the pyrrolidine ring in preussin (Figure 1) when compared to preussin C (without the *N*-methyl group on the pyrrolidine ring) on antibacterial and cytotoxic activities [23].

Most in vitro studies with cells have used the two-dimensional (2D) culture method, for which the cells grow in a monolayer and adherent to a solid substrate [4]. Despite its advantages, such as the simplicity of methods and the low costs associated with maintaining cell cultures and performing research tests, cultures with adherent cells have disadvantages that limit their use and the conclusions drawn from them [4,45]. The disposition of the cells as a monolayer provides them a homogeneous exposure to the culture medium, resulting in similar availability of oxygen and nutrients to the cultured cells. Furthermore, the disposition of the cells also influences the cell morphological characteristics resulting in an altered cell structure, which is more elongated and flattened. It also affects the cell–cell interactions and cell junctions, which are decreased in the 2D cell culture model. These altered characteristics result in modified signaling pathways, leading to altered cell metabolism, differentiation and proliferation rates, viability, cell invasion, transcriptional and apoptotic patterns, and sensitivity to different compounds, not mimicking the tissue and tumor structure, behavior, and microenvironment complexity found in vivo [4,45,46,47,48,49]. Therefore, in the quest for anticancer drugs, the in vitro effects of bioactive compounds observed in 2D cultures should be refined with more complex approaches.

Due to its three-dimensionality, the 3D cell culture model (forming multicellular aggregates—MCAs) shows important features for anti-cancer drug discovery and development, including the capability to mimic tumor characteristics in vivo such as cell morphology, gene expression, cell surface receptor organization, drug resistance, hypoxia, migration, proliferation, differentiation, dormancy, and anti-apoptotic patterns, as well as the cell-to-cell interaction and the tumor microenvironment including cell–extracellular matrix interactions [45,48,50].

In this study, we expanded the knowledge of biological properties and effects of preussin in the TNBC cell line, MDA-MB-231, by comparison of the results in the 2D and 3D cell culture models. We first selected the lowest concentrations of preussin that decrease cell viability (both in 2D and 3D cell cultures) [43]. With these concentrations, antiproliferative, genotoxic, and cytotoxic (distinguishing between apoptosis and necrosis) properties of preussin and ultrastructural morphological alterations were then evaluated in the 2D and 3D cell culture models. Furthermore, the anti-migratory properties of preussin were tested in the 2D model. Thus, the major aim of our study is to offer new data to support a better assessment of preussin as a potential molecule for anti-cancer drug development.

## 2. Results

### 2.1. Assessment of Cell Viability—MTT Assay

#### 2.1.1. Monolayer Cell Culture

In 2D cell culture, the effects of preussin on cell viability in the TNBC cell line, MDA-MB-231, were assessed by the MTT assay after 24, 48, and 72 h of incubation.

**Figure 2:**
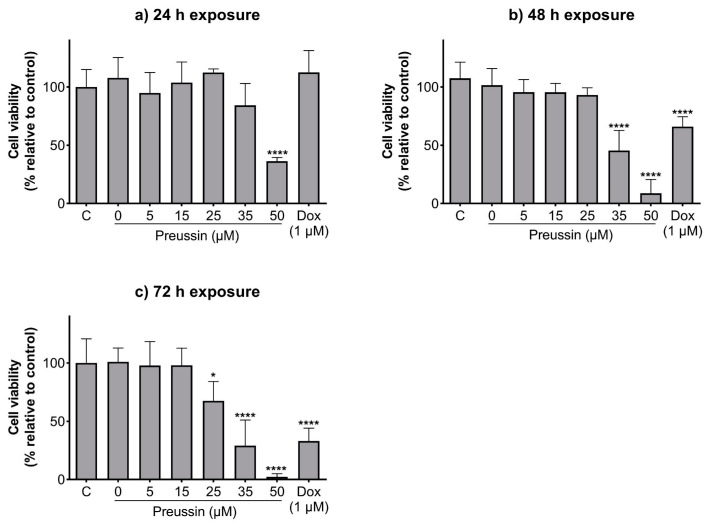
Effects of preussin on cell viability of MDA-MB-231 cells in monolayer cell culture assessed by the MTT assay after 24 h (**a**), 48 h (**b**), or 72 h (**c**) of exposure. Negative control (C) (cells in cell culture medium), solvent control (0 µM of preussin and 0.1% of DMSO), and the positive control (cells exposed to 1 µM of Dox) groups were included. The results are expressed as the percentage of cell viability relative to the negative control and are expressed as mean ± SD of *n* = 5. Significant differences, presented with asterisks (* *p* < 0.05; **** *p* < 0.0001), were tested by one-way ANOVA, followed by the post-hoc Holm–Šidak’s test.

Preussin showed an increase in its effect on cell viability over time (Figure 2). Cells exposed to preussin at 50 µM showed a decrease in cell viability even at 24 h of exposure, with a reduction in cell viability to 36.4% in relation to the negative control. At 35 µM, preussin decreased cell viability compared to the negative control at times of exposure above 48 h. At 48 h of exposure, preussin at 35 µM reduced cell viability below 45.5%, and at 72 h the cell viability was 29.1% (compared to the negative control group) (Figure 2b,c). At 72 h of exposure, 25 µM of preussin was the lowest concentration to significantly decrease the cell viability to 67.5%, compared to the negative control (Figure 2c). Similar to what was observed for preussin at 35 µM, doxorubicin (Dox) at 1 µM only exhibited decreased cell viability with incubation times above 48 h, showing cell viability of 65.9% and 33.1% at 48 h and 72 h of exposure, respectively (Figure 2b,c, respectively). When analyzing the dose–response curves of cells exposed to preussin for 72 h (Figure S1a), the IC_50_ value was 30.06 (at a confidence interval of 95%: 25.31 to 81.13), while the dose–response curve (Figure S1b) analysis from our prior assays [43] gave an IC_50_ value of 39.18, differing 26.3% from the IC_50_ value of the present study. There were no statistically significant differences between the negative control and the solvent groups at all exposure times (Figure 2).

#### 2.1.2. Multicellular Aggregates (MCAs)

The effects of preussin at 25, 35, and 50 µM (the concentrations that decreased cell viability in 2D culture after 72 h of exposure) on cell viability in MDA-MB-231 cells, cultured as MCAs, were assessed by the MTT assay after 96 h of incubation.

**Figure 3:**
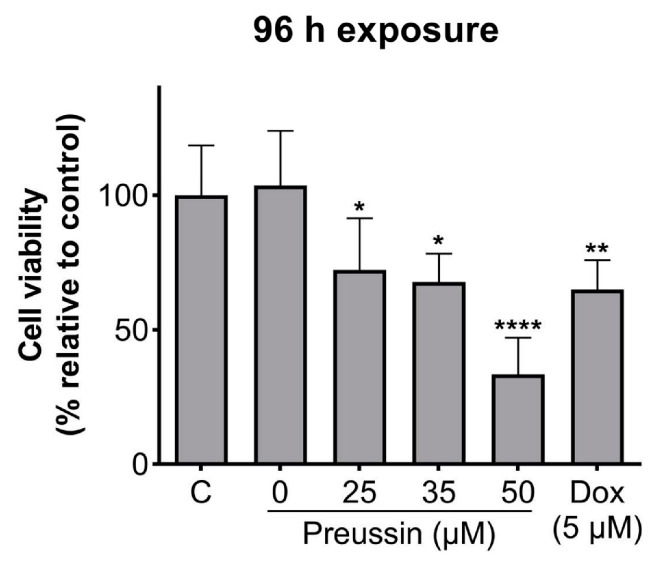
Effects of preussin on cell viability in MDA-MB-231 cells, cultured as MCAs, assessed by the MTT assay after 96 h. Negative control (C) (cells in cell culture medium), solvent control (0 µM of preussin and 0.1% of DMSO), and positive control (cells exposed to 5 µM of Dox) groups were included. The results are expressed as the percentage of cell viability relative to the negative control and are expressed as mean ± SD of *n* = 5. Significant differences, presented with asterisks (* *p* < 0.05; ** *p* < 0.01; **** *p* < 0.0001), were tested by one-way ANOVA, followed by the post-hoc Holm–Šidak’s test).

With 96 h of exposure, all the tested concentrations of preussin showed decreased cell viability when compared to the control, as shown in Figure 3. This decrease was higher at 50 µM of preussin, where the observed cell viability was 33.4%. At 25 and 35 µM of preussin and 5 µM of Dox, the decrease in cell viability was similar, with viability calculated values of 72.3%, 67.7%, and 65%, respectively (Figure 3). Based on these results, the concentrations of 25 and 35 µM of preussin were selected to explore further mechanisms. At 24 h of exposure, none of these groups decreased cell viability when compared to the negative control group (Figure S2). Statistically significant differences were not found between the negative control and the solvent control groups (Figure 3).

### 2.2. Effects of Preussin on Cell Proliferation—5′-bromo-2′-deoxyuridine (BrdU)

**Figure 4:**
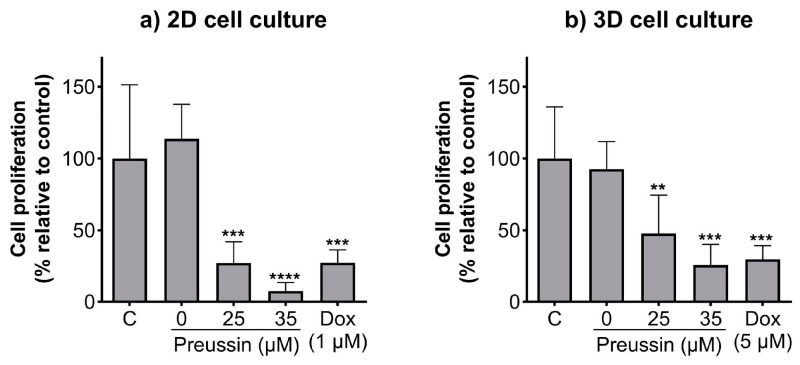
Effects of preussin on cell proliferation of MDA-MB-231 cells, cultured as a monolayer and exposed for 72 h (**a**) or cultured as MCAs and exposed for 96 h (**b**), assessed by the BrdU assay. Negative control (C) (cells in cell culture medium), solvent control (0 µM of preussin and 0.1% of DMSO), and positive control (cells exposed to 1 µM (2D cell culture) or 5 µM (3D cell culture models) groups were included. The results are presented as the percentage of cell proliferation relative to the negative control and are expressed as mean ± SD of *n* = 5. Significant differences, presented with asterisks (** *p* < 0.01; *** *p* < 0.001; **** *p* < 0.0001), were evaluated by one-way ANOVA, followed by the post-hoc Holm–Šidak’s test.

The BrdU assay was used to evaluate whether the effects of preussin on cell viability in the 2D and 3D cell culture models resulted from alterations in cell proliferation. The lower concentrations of preussin that affected cell viability (25 and 35 µM) were selected to evaluate their effects on cell proliferation. Cells cultured in monolayer and MCAs were exposed to 25 or 35 µM of preussin for 72 h (monolayer) or 96 h (MCAs). In both cell culture models, all the tested concentrations of preussin induced a decrease in cell proliferation, which was more pronounced in the cells in the 2D cell culture model (Figure 4). Preussin, at 25 and 35 µM, significantly decreased cell proliferation in the cells, cultured in monolayer, to around 27.1% and 7.6% (compared to the negative control), respectively (Figure 4a). As for the cells cultured in the 3D cell culture model, preussin showed a slightly lower effect in decreasing cell proliferation. Compared to the negative control group, the MCAs exposed to 25 µM of preussin showed a decrease in cell proliferation to 47.6%, while 35 µM of preussin decreased cell proliferation to 25.7% (Figure 4b). No statistically significant differences were observed in cell proliferation between the solvent and the negative control groups. As for the positive control, in the 2D and 3D cell culture models, Dox (at 1 µM in monolayer, and 5 µM in MCAs) induced similar effects with a decrease in cell proliferation to below 30% when compared to the negative control groups.

### 2.3. Analysis of Cell Migration—In Vitro Scratch Assay

**Figure 5:**
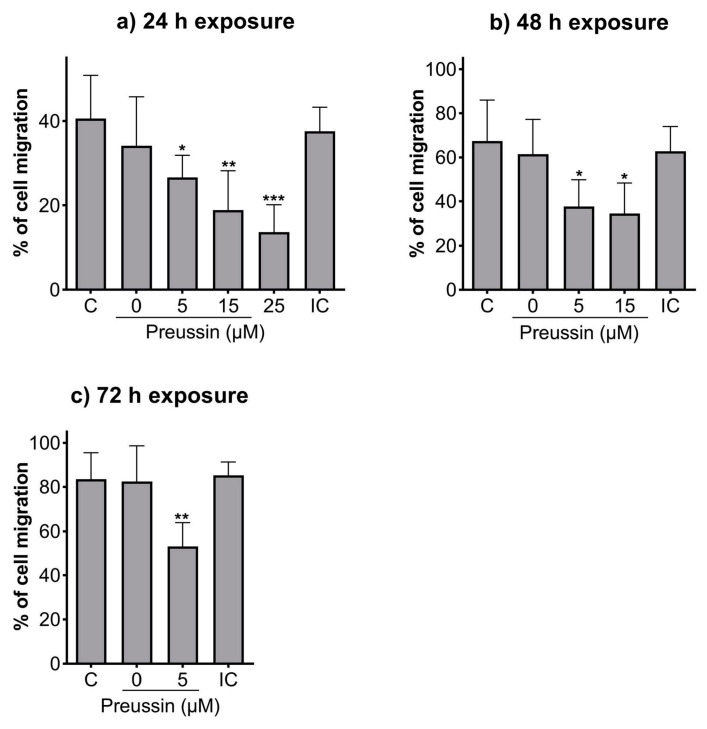
Effects of preussin on cell migration of MDA-MB-231 cells, cultured as a monolayer after 24 h (**a**), 48 h (**b**), or 96 h (**c**) of exposure, assessed by the in vitro scratch assay. Negative control (C) (cells in DMEM supplemented with 1% fetal bovine serum (FBS), solvent control (0 µM of preussin and 0.1% of DMSO), and internal control (IC) (cells exposed to DMEM supplemented with 10% FBS). The percentages of cell migration are expressed as mean ± SD of *n* = 5 (three replicates per experiment). Significant differences, presented with asterisks (* *p* < 0.05; ** *p* < 0.01; *** *p* < 0.001), were evaluated by one-way ANOVA, followed by the post-hoc Holm–Šidak’s test.

Compared to the negative control, all the tested concentrations of preussin could decrease cell migration at all the tested time points (Figure 5). After 24 h of exposure, cells exposed to preussin at 35 µM appeared floating (at a considerably higher number than that observed for the control groups), and after 48 h, all the cells were completely detached from the bottom of the plate (characteristics suggestive of cell death), making it unfeasible to measure the scratches (Figure S3). Whenever the characteristics indicating increased cell death (cell detachment, cell rounding up, and/or granular morphology) were observed, no measurements were taken to exclude the confounding factor of cell death. At 24 h of exposure, preussin at 25 µM decreased cell migration compared to the solvent control. The effect of preussin on cell migration was accentuated at the concentrations of 5 µM and 15 µM, with higher exposure times. At 25 µM of preussin and after 48 h of exposure, characteristics suggesting high levels of cell death were observed, with cells detached from the bottom of the plate. Such death and detachment were also observed for cells exposed to preussin at 15 µM for 72 h (Figure S3). For this reason, no measurements of the scratches were made for cells exposed for 48 h or 72 h to preussin at 25 µM or exposed to preussin at 15 µM for 72 h (Figure 5). After 24 h, the exposure to 5 µM of preussin decreased cell migration by 13.9%, the exposure to 15 µM of preussin decreased cell migration by 21.7%, and the exposure to 25 µM of preussin decreased cell migration by 26.9% when compared to the negative control group (Figure 5a). After 48 h of exposure, there was an increase in the effects of preussin on cell migration. While the measured migration ratio in the negative control was 67.4%, in the cells exposed to preussin at 5 µM and 15 µM, the measured migration decreased to 37.6% and 34.6%, respectively (Figure 5b). After 72 h of exposure, the negative, solvent, and internal controls showed almost a complete closure of the scratches (cell migration above 80%). As for the cells exposed to 5 µM of preussin, a decrease of more than 30% in cell migration was observed compared to the negative control group, with a measured wound closure of 53.1% (Figure 5c). No statistically significant differences were observed among the control groups at any of the evaluated time points (Figure 5).

### 2.4. Detection of DNA Damages—Comet Assay

**Figure 6:**
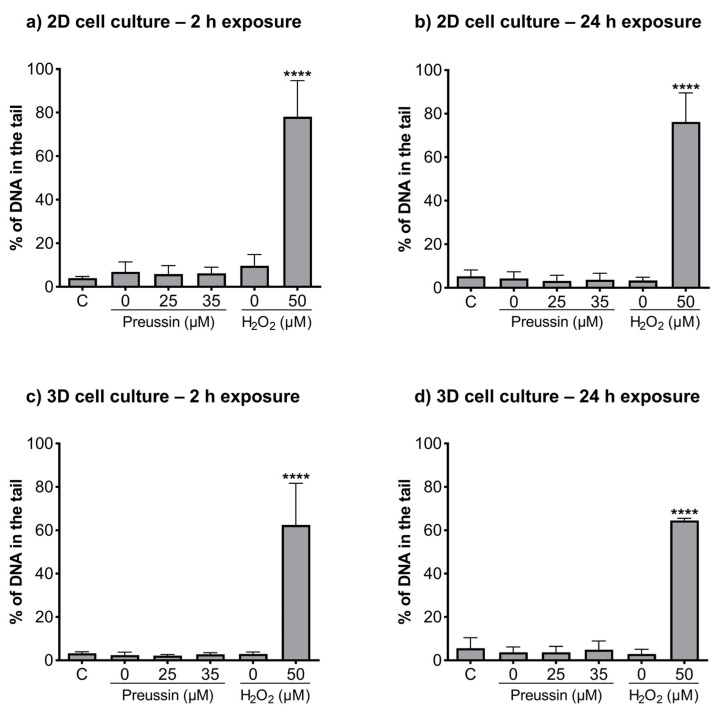
Genotoxic effects (induction of strand breaks) of preussin at 25 and 35 µM on MDA-MB-231 cells cultured in the 2D cell culture model after 2 h (**a**) or 24 h (**b**) of exposure or cultured in the 3D cell culture model and exposed for 2 h (**c**) or 24 h (**d**), measured by a standard version of the comet assay. The negative control (C) consisted of cells exposed only to the cell culture medium, the solvent control (0 µM of preussin) of cells exposed to 0.1% (*v*/*v*) of DMSO, and the positive control of nucleoids exposed to 50 µM of H_2_O_2_ in PBS. The results were expressed as the percentage of DNA in the comet’s tail. The results are expressed as mean ± SD of *n* = 5 (from 50 comets per gel and 2 gels per condition). Significant differences from the negative control group, presented with asterisks **** *p* < 0.0001), were evaluated by one-way ANOVA, followed by the post-hoc Holm–Šidak’s test.

**Figure 7:**
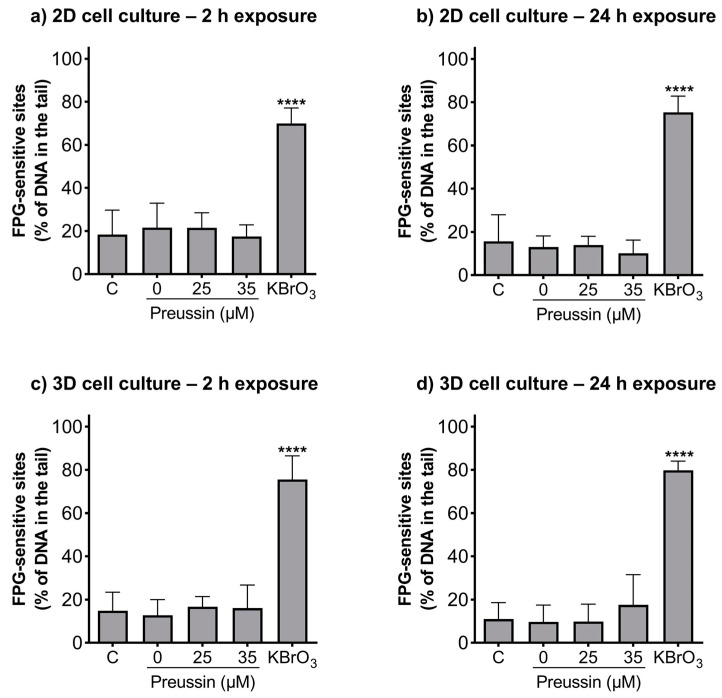
Genotoxic effects (FPG sensitive sites) of preussin at 25 and 35 µM on MDA-MB-231 cells cultured in the 2D cell culture model after 2 h (**a**) or 24 h (**b**) of exposure or cultured in the 3D cell culture model and exposed for 2 h (**c**) or 24 h (**d**). Negative control (C) (cells exposed only to cell culture medium), solvent control (0 µM of preussin) (cells exposed to 0.1% (*v*/*v*) DMSO), and positive control (cells exposed for 1 h to 4.5 mM of KBrO_3_) groups were selected. The results were expressed as the percentage of DNA in the tail of the comet and calculated by subtracting the percentage of DNA damage of the non-FPG enzyme-treated samples from the FPG enzyme-treated samples. The results are expressed as mean ± SD of *n* = 5 (from 50 comets per gel and 2 gels per condition). Significant differences, presented with asterisks **** *p* < 0.0001), were evaluated by one-way ANOVA, followed by the post-hoc Holm–Šidak’s test.

The alkaline and formamidopyrimidine DNA glycosylase (FGP) versions of the comet assay were used to evaluate if preussin had genotoxic effects as well as to identify the type of induced DNA damage. In both the MCAs and cells cultured in monolayer, DNA damage was analyzed after exposure to non-cytotoxic concentrations of preussin (viability higher than 70%), assessed by both the MTT (Figure 2 and Figure S2) and trypan blue exclusion assays (Figure S4). At the tested concentrations (25 and 35 µM), preussin did not induce DNA damage, either by strand breaks (Figure 6) or oxidative damage (FPG sensitive sites) (Figure 7). The results were similar in both 2D and 3D cell cultures.

### 2.5. Induction of Apoptosis—Annexin V—PI Assay

The annexin V-PI assay was used to evaluate the effects of preussin in the induction of apoptosis in MDA-MB-231 cells. This assay detects membrane changes that occur during the apoptotic process (translocation of the phosphatidylserine residues, to which annexin-V protein binds, from the inner to the outer leaflet of the cell membrane) and allows its distinction from necrosis and late apoptosis (where membrane disruption allows entry of propidium iodide (PI) and binding to the cell DNA) [51].

**Figure 8:**
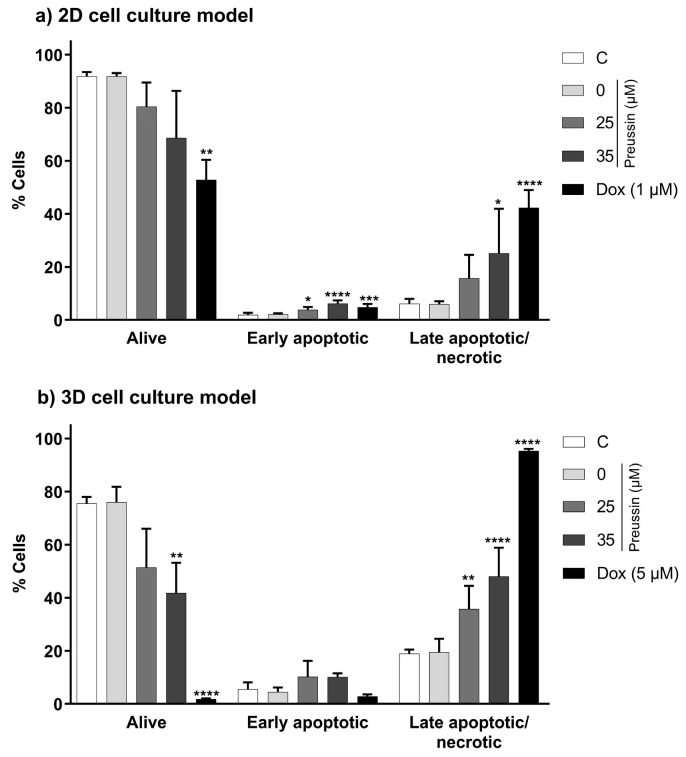
Effects of preussin at 25 and 35 µM, in the induction of cell death (late apoptosis/necrosis) and early apoptosis, on MDA-MB-231 cells, cultured in the 2D (**a**) or 3D (**b**) cell culture models, respectively, exposed for 72 h or 96 h. Early apoptosis and late apoptosis/necrosis induction were assessed by flow cytometry upon cell Annexin V-FITC/PI double staining, followed by analysis with the FlowJo^TM^ software. The negative control (C) consisted of cells exposed only to the cell culture medium, the solvent control (preussin at 0 µM) of cells exposed to 0.1% (*v*/*v*) DMSO, and the positive control of cells exposed to Dox (at 1 µM in 2D cell culture and 5 µM in 3D cell culture). The results were expressed as the percentage of cells in each state, either viable, in early apoptosis, or in late apoptosis/necrosis. The results are expressed as mean ± SD of *n* = 5. Statistically significant differences between the percentage of cells in each biological state (viable, early apoptotic, or late apoptotic/necrotic) in each exposure group, and the percentage found in the negative control group are presented with asterisks (* *p* < 0.05; ** *p* < 0.01; *** *p* < 0.001; **** *p* < 0.0001). Differences were evaluated by one-way ANOVA, followed by the post-hoc Holm–Šidak’s test (in the early apoptotic and late apoptotic/necrotic cells groups) or the Brown–Forsythe and Welch ANOVA tests (for the living cells).

In the 2D cell culture model, only preussin at 35 µM increased the percentage of cells in the late apoptosis/necrosis state compared to the negative control group, with an increase of 19% (Figure 8a). As for the 3D cell culture model, preussin at 25 and 35 µM increased the rate of cells in late apoptosis/necrosis compared to the negative control group, with an increase of 16.9% and 29.1%, respectively (Figure 8b).

When evaluating an early apoptosis induction, differences between the tested concentrations of preussin (25 and 35 µM) and the negative control were only observed in the cells in the 2D cell culture model (Figure 8). In the 2D cell culture, cells exposed to preussin at 25 and 35 µM showed an increase of 1.9% and 4.3%, respectively, when compared to the negative control (Figure 8a). Although statistically significant, this increment in the percentage of early apoptotic cells was much lower than that observed for the percentage of the cells in the late apoptosis/necrosis.

No statistically significant differences were observed between the negative and solvent control groups in the functional statuses (alive, late apoptotic/necrotic, or early apoptotic) of the cells from 2D and 3D cell cultures. As for the positive control group, Dox increased the percentage of cells in late apoptosis/necrosis, both in cells from 2D and 3D cell culture models, and slightly increased the proportion of cells in early apoptosis in the monolayer cultures (when compared to the respective negative control groups) (Figure 8).

When compared between the 2D and 3D cell culture models, the percentage of cells in late apoptosis/necrosis was higher in the cells cultured as MCAs (negative, solvent, and positive controls and the preussin-exposed groups) than in the 2D cell culture model (Figure 8).

### 2.6. Effects of Preussin on Cell Morphology

#### 2.6.1. Phase-Contrast Microscopy and Stereomicroscopy

Over time, we observed a decrease in cell density compared to the negative and solvent control groups in cells exposed to preussin. Cell rounding up, decreased substrate adherent cells, and the appearance of cell debris were identified after 24 h of exposure to preussin at 50 µM. After 48 h of exposure, cell morphology alterations occurred at lower concentrations when compared to the solvent and negative controls (Figure S5).

No apparent visual differences were observed between the negative and solvent control groups and the MCAs exposed to 5, 15, 25, 35, and 50 µM of preussin groups (Figures S6 and S7). The MCAs exposed to 5 µM of Dox showed slightly higher diameter (Figures S6 and S7), less density, and were more flattened than those in the negative and solvent control groups. Accordingly, the MCAs exposed to Dox disaggregated more easily, even with gentle manipulation. No statistically significant differences were found between the area of MCAs from the negative control and the other exposure groups (Figure S7).

#### 2.6.2. Transmission Electron Microscopy (TEM)

Cells cultured in monolayer and MCAs were exposed to preussin (for 72 h and 96 h, respectively) at concentrations of 5, 15, 25, or 35 µM to assess the ultrastructural effects. As a negative control, cells in monolayer and MCAs were incubated only with DMEM, as a solvent control, cells were incubated with 0.1% (*v*/*v*) DMSO, and as a positive control, cells were exposed to Dox at 1 or 5 µM. After exposure, cells in monolayer and MCAs were observed with TEM.

**Figure 9:**
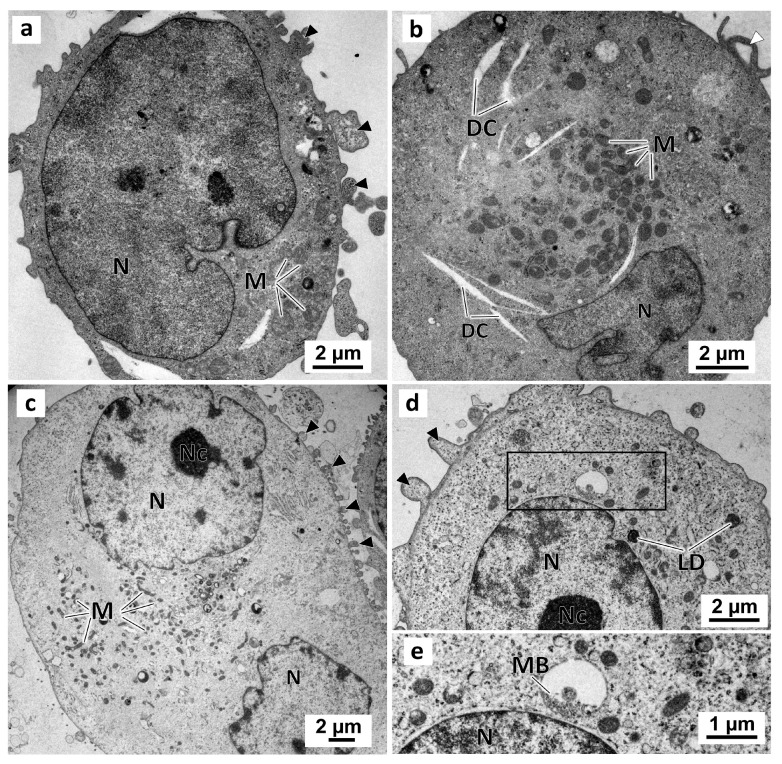
Representative images of TEM analysis of MDA-MB-231 cells cultured as a monolayer. Cells were exposed for 72 h only to DMEM, in the negative control (**a**,**b**), or to 0.1% (*v*/*v*) DMSO, in the solvent control (**c**,**d**). Detail of the enclosed area in (**d**) showing the multivesicular bodies. Black arrowheads: membranes blebs; White arrowheads: slender filopodia-like structures; DC: dilated cisternae; LD: lipid droplets; M: mitochondria; MB: multivesicular bodies; N: nucleus; Nc: nucleolus.

No morphological differences were observed between cells from the negative and solvent control groups (Figure 9) in the monolayer culture. In these two groups, cells presented irregular-shaped nuclei with prominent nucleoli (Figure 9c,d). Moreover, there were abundant mitochondria (Figure 9a–c), some lipid droplets, and multivesicular bodies (Figure 9d,e) in the cytoplasm. The cell surface evidenced membrane blebbing (Figure 9a,c,d) and filopodia-like structures (Figure 9b).

**Figure 10:**
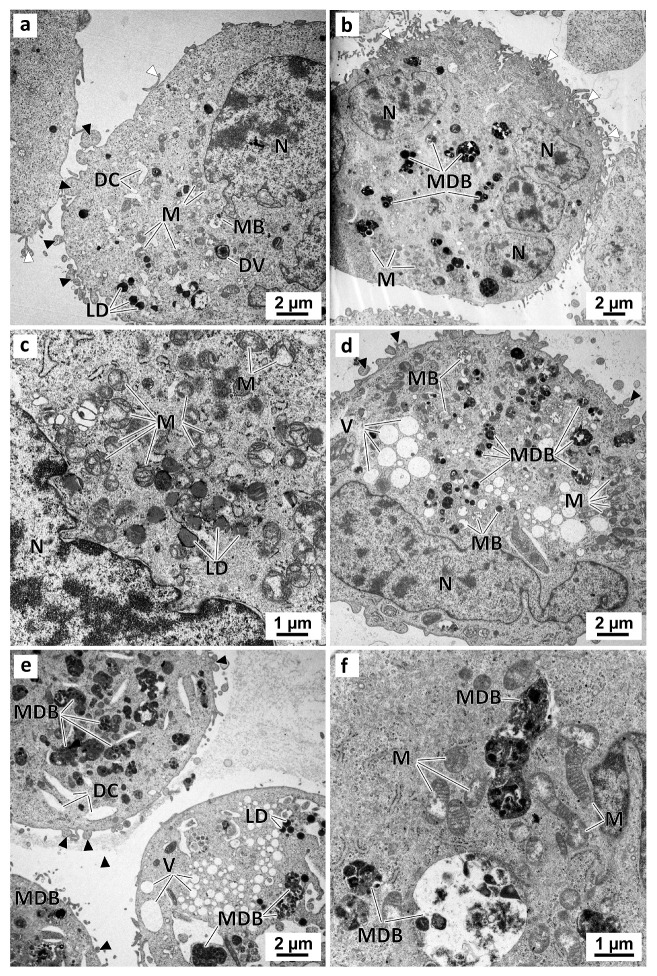
Representative images of TEM analysis of preussin-exposed MDA-MB-231 cells cultured as a monolayer. Cells were exposed for 72 h to several concentrations of preussin: 5 µM (**a**), 15 µM (**b**,**c**), 25 µM (**d**), and 35 µM (**e**,**f**). Black arrowheads: membranes blebs; White arrowheads: slender filopodia-like structures; DC: dilated cisternae; DV: degenerative vesicles; LD: lipid droplets; M: mitochondria; MB: multivesicular bodies; MDB: multivesicular electron-dense bodies; N: nucleus; NC: nucleolus, V: vesicles.

In the preussin-treated cells, the ultrastructural alterations were dose-dependent. With 5 µM of preussin (Figure 10a), there were no significant visual differences compared to the control groups. With increasing concentrations of preussin, above 15 µM, a higher number of pleomorphic multivesicular bodies of different sizes were observed, some with electron-dense bodies inside (Figure 10b,d–f) (Figure S8a). Similarly, more enlarged cytoplasmic vesicles were observed (Figure 10d,e). The number and pleomorphism of the larger cytoplasmic vesicles and multivesicular bodies (mainly the multivesicular bodies containing electron-dense material) highly increased with the concentration of preussin. Furthermore, with preussin concentrations above 15 µM, mitochondria exhibited considerable damage, with their cristae becoming more structurally disordered (Figure 10c) and faded (Figure 10f).

**Figure 11:**
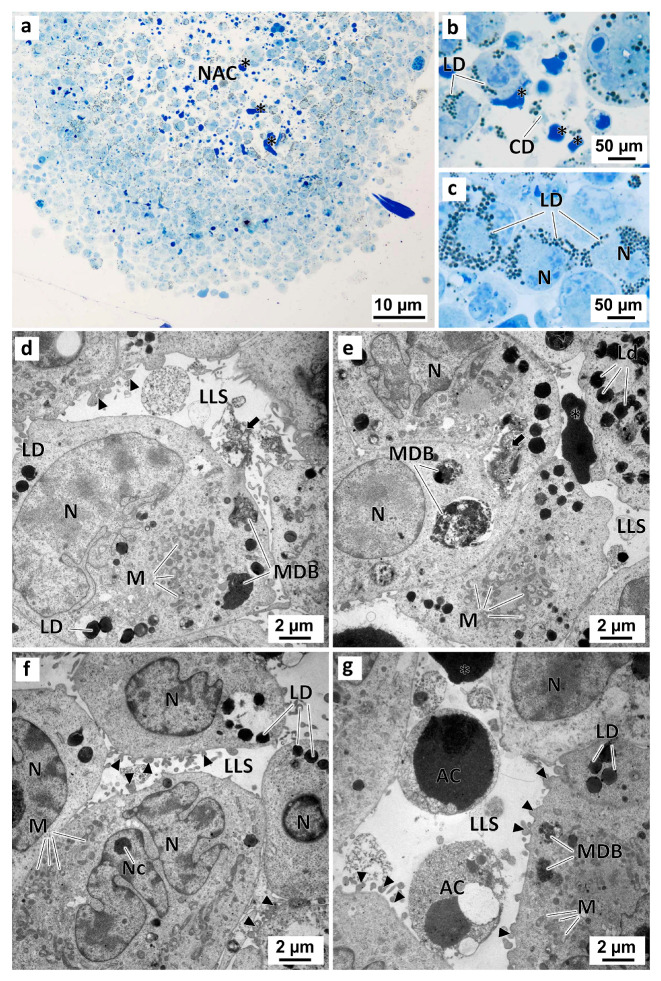
Representative images of semithin sections (**a**–**c**) and TEM (**d**–**g**) analysis of MDA-MB-231 MCAs. The MCAs were exposed for 96 h only to DMEM in the negative control (**a**–**e**), or to 0.1% (*v*/*v*) DMSO, in the solvent control group (**f**,**g**). Asterisk: electron-dense amorphous material; black arrows: electron-dense slightly granular material; black arrowheads: membranes blebs; AC: cells with compatible apoptotic morphology; CD: cell debris; LD: lipid droplets; LLS: lumen-like structures; M: mitochondria; MDB: multivesicular dense bodies; NAC: necro-apoptotic core; N: nucleus; Nc: nucleolus.

The MCAs cells displayed both similarities and differences from the patterns found in the monolayer cultured and exposed cells. Similar to the 2D cell cultures, no relevant morphological differences were observed between the MCAs in the negative control group and those in the solvent control group (Figure 11). Irrespective of that, the analysis of semi-thin sections revealed that some of the MCAs had a necrotic-apoptotic core filled with dead cells, cell debris, and dense intercellular material (Figure 11a,b). Under TEM, the MCAs from the control groups had irregular-shaped nuclei and well-developed organelles (mainly mitochondria) (Figure 11d–g). In contrast to the observations in the 2D cell culture model, the cells from MCAs displayed a significantly higher quantity of lipid droplets in the cytoplasm (Figure 11c,e,f) (Figure S8b) and some pleomorphic multivesicular electron-dense bodies (Figure 11e,f) (Figure S8a). MCAs from the control groups also showed high cell-to-cell adhesion, with cells connecting in several regions alongside the cellular membranes. It was also possible to observe the formation of lumen-like structures, among cells, with membrane blebbing and short filopodia-like projections on the cell surface (Figure 11d,g). Furthermore, these lumen-like structures were sometimes filled with electron-dense material, either amorphous or slightly granular (Figure 11d,e). In some of these luminal structures, it was possible to observe necrotic cells, evidencing shrinkage, nuclear pyknosis, and some degree of cytoplasmic vacuolization, and apoptotic shrinking cells, with chromatin condensation and preservation of cell integrity and membranes (Figure 11g).

**Figure 12:**
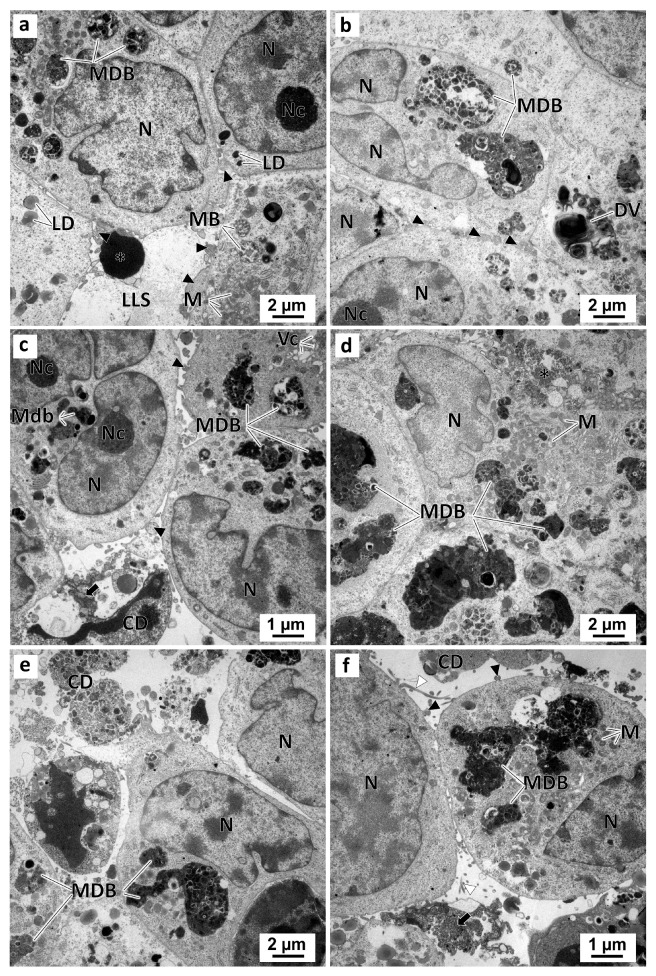
Representative images of TEM analysis of preussin-exposed MDA-MB-231 MCAs. The MCAs were exposed for 96 h to preussin at concentrations of 5 µM (**a**), 15 µM (**b**), 25 µM (**c**,**d**), and 35 µM (**e**,**f**). Asterisk: electron-dense amorphous material; black arrows: electron-dense, slightly granular material; black arrowheads: membranes blebs; CD: cell debris; DV: degenerative vesicles; LD: lipid droplets; LLS: lumen-like structures; M: mitochondria; MB: multivesicular bodies; MDB: multivesicular dense bodies; N: nucleus; Nc: nucleolus.

In the preussin-exposed MCAs, ultrastructurally morphological alterations occurred in a dose-dependent manner. With the increase in preussin concentration, the number of cells having pleomorphic multivesicular structures with electron-dense bodies inside increased (Figure S8a). The higher the preussin concentration, the greater the pleomorphism of these multivesicular dense bodies and degenerative vesicles (Figure 12). Moreover, with increasing preussin levels, the amorphous or slightly granular electron-dense material emerged in the lumen-like structures. Accordingly, more cell debris, a higher number of necrotic cells (with cells losing the organelles and membrane rupture), and apoptotic cells (with cell shrinkage, pyknosis, and DNA condensation) were also observed in those lumen-like structures. Finally, higher concentrations of preussin led to less cell aggregation (Figure 12). Accordingly, there were more cell debris, a higher number of necrotic cells with organelle loss, and membrane rupture. Apoptotic cells were also observed in the lumen-like structures with cell shrinkage, pyknosis, and DNA condensation. Moreover, less cell aggregation was observed with the increase in preussin concentration (Figure 12).

## 3. Discussion

TNBC is an aggressive subtype of breast cancer, characterized by an invasive nature, rapid disease progression, low survival, and high recurrence rates. Furthermore, it has few therapeutic options [52,53,54,55]. This study elucidated the in vitro anticancer activities of the marine fungus-derived compound, preussin, in a TNBC cell line, MDA-MB-231, both in 2D culture and in a more representative model of the tumor behavior in vivo, the 3D cell culture model. We confirmed the antiproliferative and cytotoxic effects of preussin in the TNBC in vitro culture model and described, for the first time, its inability to cause genotoxic effects. We further characterized the preussin-induced ultrastructural changes, a frequently neglected type of analysis. To the best of our knowledge, this is the first study of its anti-migratory effects. Finally, we further compared the differences between the 2D and 3D cell culture models used for testing preussin and Dox (reference control).

Malhão et al. [43] have previously tested the effects of preussin in a set of BC cell lines, including SKBR3, MCF-7, and MDA-MB-231 (each of which represents a different clinicopathological surrogate subtype of BC), and a non-tumoral breast cell line, MCF-12-A, observing a cytotoxic effect of preussin at 50 µM, for all tested cell lines cultured in the 2D cell culture model. However, the MDA-MB-231 cell line showed a higher resistance when compared to other BC cell lines. MDA-MB-231 is a mesenchymal-stem-like BC adenocarcinoma cell line, characterized by the lack of expression of ER, PGR, and HER2 markers, which classifies it as a TNBC cell line [56,57]. MDA-MB-231 represents an invasive and aggressive subtype of BC for which limited therapeutic options exist, especially when compared to other BC subtypes. Therefore, MDA-MB-231 cells were selected as a proxy to evaluate the properties of preussin. In the present study, over time, the MDA-MB-231 cells cultured in the 2D cell culture model showed a gradual decrease in cell viability after being exposed to preussin, in a dose-dependent manner.

The use of the in vitro 3D cell culture model to study the effects of preussin in cell culture has been reported only once by Malhão et al. [43] who demonstrated that different breast cancer cell lines, including MDA-MB-231, displayed higher resistance to preussin-induced reduction in cell viability in the 3D cell cultures. In this study, the lowest concentration of preussin that showed cytotoxic effects in a monolayer cell culture also induced cytotoxicity in the 3D cell culture model. It is important to note, however, that even though the tested concentrations of preussin were the same in the two culture models (25 and 35 µM), the times of exposure to preussin were different (72 h in the 2D cell culture model against 96 h in the 3D cell culture model). Therefore, our findings indirectly suggest that MDA-MB-231 cells are more resistant to preussin in 3D than in 2D cell cultures when analyzing cell viability, but cells can still be affected in 3D cell culture by using longer exposures.

According to the literature, different cell lines in 3D cell culture can display either higher resistance or higher sensitivity to drugs when compared to cells in 2D cell culture [58,59,60]. For the MDA-MB-231 line, the current data revealed that 3D cultured cells have higher resistance to Dox than those in 2D culture [61,62].

The 3D architecture of MCAs may (at least partially) explain the higher drug resistance compared to 2D cell culture, possibly due to the differences in drug penetration between the 2D and 3D cell cultures [63]. Therefore, the MDA-MB-231 MCAs architecture and the cell cohesiveness may reduce preussin and Dox penetration, resulting in a lower bioavailability of the compounds to all cells. It is important to note, however, that different authors have described the obtention of MDA-MB-231 cell line 3D structures with varying levels of cohesiveness [58,60,64,65,66,67,68,69] and varying resistance to drugs (such as Dox) when we compared 2D with 3D cell cultures [60,64]. Malhão et al. [65], using the same cell line and method to obtain the 3D culture, have found the MCAs of MDA-MB-231 with an ellipsoid form while maintaining cohesive 3D structures. In this study, the existence of cell adhesions, as observed in the ultrastructural analysis of these MCAs, supports the previous findings [65] and further confirms the cohesiveness of the MDA-MB-231 MCAs.

Other factors have been proposed for the higher resistance of 3D cultures compared to that of 2D cultures. Lovitt et al. [62] correlated the lower cytotoxicity of Dox in 3D cell cultures with the lower proliferation rate observed in this cell culture model compared to the 2D cell culture. This lower proliferation rate may result in a lower sensitivity to drug cytotoxicity compared to cells with higher doubling times [62]. Another resistance mechanism that happened in MDA-MB-231 3D cultures is the up-regulation of the pro-survival proteins [62].

Hypoxia and pH may also contribute to resistance mechanisms. In spheroids, as described for solid tumors, the inner cells have lower oxygen availability, dependent on its diffusion throughout the spheroid/tumor and its consumption by the outer cells [70]. This differential accessibility induces hypoxia in the inner cells which, in turn, may induce the expression of molecules responsible for external drug export, the activation of anti-apoptotic factors, the expression of growth factors, and the inhibition of oxidative stress due to the inexistence of oxygen [71]. Furthermore, oxygen-deprived cells produce lactate, which decreases the pH of the inner portion of the spheroid [71]. Low pH values may affect drug action by impairing the uptake of some drugs, as occurs with Dox [71].

Regarding the comparability of the variable results described in the literature for the preussin-induced decrease in cell viability, it is important to mention that all the methods used were different, relying on diverse proxies of viability, which may lead to different conclusions. In the present study, as in many others [23,43], the MTT assay was used to evaluate the effects of preussin on cell viability. Achenbach et al. [42] calculated the cell viability by measuring the crystal violet dye retained by cells after 48 h of exposure to preussin, followed by incubation in a drug-free medium for another 48 h. Malhão et al. [43] have shown that the evaluation of cytotoxicity of preussin in 2D and 3D cell cultures model, assessed by multiple assays (including MTT, LDH, and resazurin reduction assays), rendered different results since several targets of cell viability (either cell metabolism or cell membrane integrity) were evaluated. Despite variability, including technicalities issues (e.g., cell passage number), the IC_50_ estimated with the data obtained in this study was reasonably comparable with that obtained from the trials in the scope of previous experiments [43], showing a difference of about 26%.

Furthermore, we also observed that with serum-deprived and serum-reduced conditions (in an attempt to decrease cell proliferation, which may appear as a confounding factor in the migration assays), MDA-MB-231 cells displayed a higher sensitivity to preussin. Lower concentrations of preussin that had not previously decreased the cell viability (in normal serum culture conditions) induced aspects of cell death (cell detachment and alteration of cell morphology) in cells exposed to serum-deprived and reduction conditions.

The serum contains growth and adhesion factors to promote cell proliferation and attachment. Mammalian cells require growth factors to enter and progress through the cell cycle [72]. Additionally, serum starvation is described to synchronize and inhibit cell proliferation in some cancer cell lines by arresting these cells at the G0/G1 phase of the cell cycle [73,74,75,76]. Furthermore, by reducing the concentration of FBS for culture maintenance, we intended to decrease cell growth and, consequently, proliferation [74,77,78]. Nonetheless, no differences existed between the cells cultured in serum-reduced or serum-complete conditions, where a similar pattern of migration and growth was observed. These may indicate either an ineffective inhibition of cell proliferation with the serum-reduced medium or the induction of a more invasive phenotype in the MDA-MB-231 cells when exposed to the serum-reduced conditions coupled with simultaneous cell proliferation in the normal serum conditions cultured cells. It was previously described that the serum-reduced medium can decrease cell proliferation in some cancer cell lines [74]. Furthermore, Ye et al. [79] reported that MDA-MB-231 cells display a more aggressive phenotype, with higher invasiveness, in a phospholipase D2-dependent manner when serum-deprived. In some cancer cell lines, serum depletion is found to increase the resistance of cancer cells to oxidative stress [74]. These authors also suggested that serum deprivation may be related to in vitro multidrug- and chemoresistance by inducing a reversible proliferation arrest of cancer cells [74]. Other authors described a higher sensitivity (with lower doses of compounds causing a decrease in cell viability) of cancer cell lines to compounds when cells were cultured under serum-reduced culture conditions [80,81].

Nonetheless, we believe that more research should be conducted to compare different culture conditions (such as serum reduction versus normal serum conditions) and their effects on cell proliferation and invasiveness, elucidating the mechanisms causing scratch closure cell proliferation, cell migration, and invasiveness.

We also further investigated some cellular processes related to the decrease in cell viability caused by preussin, including cell death induction, either by necrosis or apoptosis (early or late), cell proliferation, and genotoxicity induction.

We also performed the annexin V-PI assay to identify the type of cell death mechanisms involved in a reduction in cell viability, distinguishing viable cells from cells in the early apoptosis and late apoptosis/necrosis. The assay showed that preussin decreased cell viability partially due to cell death induction. The compound induced a slight increase in the percentage of cells in the early apoptosis, mainly in MDA-MB-231 cells in 2D culture, but a significant increase in the percentage of cells in the late apoptosis/necrosis state. Interestingly, this increase in late apoptosis/necrosis induction appeared to be more significant in the 3D than in the 2D cultured cells. Indeed, the lowest tested concentration of preussin only induced an increase in late apoptosis/necrosis on the MDA-MB-231 MCAs.

Achenbach et al. [42] have previously noticed that higher concentrations of preussin increased chromatin condensation, indicating cell apoptosis induction in the HL-60 cell line. Similar to what we have observed for the TNBC cell line, MDA-MB-231, preussin has previously been shown to induce necrosis and apoptosis (through caspase-8 and cytochrome c-caspase-9 apoptotic pathways) in the HL-60 cell line. Consistently, Malhão et al. [43] described the induction of caspase-dependent apoptosis by demonstrating the expression of caspase-3 in different BC cell lines, including the MDA-MB-231, after exposure to preussin.

Apoptosis may be a secondary response to DNA damage [82]. Accordingly, we postulated that preussin could induce genotoxic effects on the TNBC cell line, MDA-MB-231, as described for *Saccharomyces cerevisiae* [44]. Since apoptosis occurs as a secondary response to DNA damage [82], and because of necrosis, such damage may also occur in the late phases [83], we tested the effects of preussin at early time points (2 and 24 h) after exposure. Nonetheless, we observed that preussin did not induce DNA damage by strand breaks, alkali-labile sites, DNA oxidative damage (oxidized purines, such as 8-oxo-7,8-dihydroguanine or formamidopyrimidines), and adducts of ring-opened N7 guanine [84], at any of the time points in any of the cell culture models, which was assessed through the alkaline and FPG modified versions of the comet assay.

In agreement with the observation that preussin induced late apoptosis/necrosis, there were signs of those phenomena in the MDA-MB-231 cells when analyzing the cell’s ultrastructure. In fact, with increasing concentrations of preussin, more cell debris (indicating a process of cell destruction) and cells with aspects of apoptosis or necrosis were observed.

With increasing concentrations of preussin, it was also possible to visualize the presence of multivesicular bodies containing electron-dense material, which increased in number and dimension. These structures were also present in higher quantities in the MDA-MB-231 MCAs than in the monolayer cultured cells, which is in agreement with the findings of other authors in this cell line [85,86]. We postulated that these membrane-bounded vesicles might be a result of cell autophagy [87,88,89]. Nonetheless, due to the high electron density of the multivesicular bodies, visualizing the double-bounding membrane typical of autophagosomes was impossible in our study. Further studies are warranted to evaluate the origin of these vesicles.

When analyzing apoptosis, necrosis, and cell survival under the influence of preussin, we noticed that the decrease in cell viability was higher than the increase in apoptosis (mainly late apoptosis) and necrosis. These results suggest that the mechanisms of action of preussin on the MDA-MB-231 cells may also occur through other processes besides cell death induction, which was confirmed by the annexin V-PI assay [90] and may include impairment of cell metabolism and proliferation.

The decrease in cell viability observed in the MDA-MB-231 cells exposed to preussin, cultured in the 3D and 2D cell culture models, was accompanied by a reduction in cell proliferation (for the two tested concentrations of 25 and 35 µM) below 50%. However, the decrease in cell proliferation was more pronounced in the cells cultured in a 2D culture model. Our data were in agreement with previous reports on the antiproliferative effects of preussin. Achenbach et al. [42] proposed that preussin inhibited cell proliferation at low concentrations but induced cytotoxicity at higher concentrations, while Malhão et al. [43] have found that preussin could inhibit MCF-7 and SKBR3 cell proliferation at concentrations above 25 µM, and it only affected MDA-MB-231 cells cultured in a monolayer at a concentration of 50 µM [43]. As for the 3D cell culture, it was observed that preussin decreased cell proliferation in all cell lines tested, at 50 µM (the lowest tested concentration) [43]. In addition, the cells exposed to preussin showed a reduced expression of the proliferation marker ki67 in BC cells cultured in a 3D culture model, including the MDA-MB-231 cell line [43].

In this study, the ultrastructural damages found in mitochondria after exposure to preussin, in a concentration-dependent manner (either disorderly or fading of the cristae), may explain not only the reduction in cell viability (since the MTT is in part metabolized by mitochondrial enzymes [91]) but also the decrease in cell proliferation (since mitochondria are involved in the regulation of the cell cycle and cell proliferation [92]). Kasahara et al. [40] reported the induction of cell cycle arrest by preussin in the G1 phase in the rat fibroblast cell line 3Y1. On the other hand, Achenbach et al. [42] have shown that the reduction in cell proliferation by preussin occurred through the inhibition of cyclin E-CDK2 kinase activity [42,93]. Consistently, cyclin E is expressed in the late G1 phase of the cell cycle and activates CDK2, which is overexpressed in TNBCs with high recurrence rates [94]. Consequently, further studies are needed to identify the molecular targets of preussin and its specificity towards cyclin E-CDK2 complex, mainly in a TNBC model, such as the MDA-MB-231 cell line.

We also tested the effects of preussin on cell migration through the scratch assay. Cell starvation and culturing in a serum-reduced medium were used to inhibit cell growth and proliferation. In further studies, we consider that it would be relevant to evaluate cell viability and proliferation to exclude the confounding factors of cell proliferation or cell death induced by exposure to the compounds. The MDA-MB-231 cell line is considered a TNBC cell line with an invasive phenotype, and it has been described that cell starvation increases the MDA-MB-231 cell aggressiveness and invasion [79,95]. Nonetheless, all the tested concentrations of preussin decreased the scratch closure, even at non-lethal doses and at short periods of exposure, suggesting a strong effect of preussin in inhibiting cell migration. Since TNBC is a cancer subtype with poor outcomes and is characterized by high rates of invasion and metastasis [52,53,54,55], developing drugs that inhibit these mechanisms is of importance. Our scratch assay data call for further studies on cell invasion and the affected pathways, including 3D strategies, such as the tumor spheroid invasion assay [96].

This study also demonstrated differences between the 2D and 3D cell culture models. In MDA-MB-231 MCAs, we detected a higher resistance to the effects of Dox and preussin, with both inducing a lower reduction in cell viability and proliferation than in the cells cultured in monolayer. Furthermore, in the unexposed cells, we observed a higher percentage of viable cells in the 2D cell culture than in the 3D cell culture. In the 3D model, we observed a slightly higher number of cells in early apoptosis and a notable difference in cells in late apoptosis and/or necrosis, compared to the 2D cell culture model. These results may correlate with the frequently spotted necrotic-apoptotic core observed in some of the MCAs evaluated by TEM. Besides the necro-apoptotic core, we discovered the presence of lumen-like structures with necrotic and apoptotic cells in the 3D cell cultures. The presence of the necro-apoptotic core in the 3D cell culture is in agreement with that described in the literature, mainly in the classically described spheroids [45,97]. Even in the new and more complex model of cell distribution within the MCAs proposed by Malhão et al. [65], it is still possible to observe a higher number of cells in the processes of apoptosis and necrosis, mainly in the center of the MCAs.

We also observed a higher accumulation of lipid droplets in the 3D cultured MDA-MB-231 cells than in the 2D cultured cells, as described previously [65]. These lipid droplets may also partially explain the different resistance of the cell culture models (3D and 2D cell cultures). Lipid metabolism involving these droplets has been associated with breast cancer malignancy and cell stemness exchanges [98]. Lipid droplets have been linked to the regulation of cell migration, metastasis, proliferation, and cell survival, all of which are involved in multidrug resistance [99,100,101]. It is well recognized that when cells are cultured in different culture models (2D or 3D), various signaling pathways are activated, and distinct patterns of cell metabolism, differentiation and proliferation rates, viability, cell invasion, transcriptional and apoptosis, as well as the sensitivity to different compounds can occur. Consequently, our current data support the idea that the 3D cell culture better mimics the tumor behavior in vivo [4,45,46,47,48,49].

The most significant properties of preussin against the TNBC cell line, MDA-MB-231, as observed in this study, include antiproliferative, cytotoxic (mainly late apoptosis/necrosis), and anti-migratory effects. Additionally, we also observed that preussin had a similar behavior on the MDA-MB-231 cell line in both 2D and 3D cultures, in a dose-dependent way.

As to future perspectives, it seems relevant to further explore the mechanism of action of preussin, to test its concomitant use with other drugs, or even to use it in drug delivery systems. In this line of thought, additional in vitro proxies should be explored to identify some of the molecular targets and signaling pathways affected by exposure to preussin. These items and avenues should include cell cycle arrest, invasion, autophagy, caspases, mitochondrial membrane potential, gene expression, and proteins related to late apoptosis/necrosis, proliferation, and migration, among others.

Finally, further studies should also be performed in non-tumoral (e.g., fibroblastic) cell lines. The results could reveal and help prevent the side effects of preussin in non-tumoral tissues, which is important to consider the use of this marine fungus-derived compound in cancer treatment. Despite the fact that the chance of any anticancer drug candidates becoming therapeutic is very slim, it is always worth exploring the mechanisms of action underlying the antiproliferative effects of preussin which could shed light on the ways this molecule interacts with TNBC cells. Even though preussin “per se” could not be a perfect lead for anticancer drug development, it can serve as a potential scaffold for further chemical modification to become an anticancer drug lead.

## 4. Materials and Methods

### 4.1. Chemicals and Reagents

Doxorubicin (D1515) (Dox), heat-inactivated FBS, collagenase from *Clostridium histolyticum*, 3-(4,5-dimethylthiazol-2-yl)-2,5-diphenyltetrazolium bromide (MTT), low melting point (LMP) agarose, normal melting point (NMP) agarose, and potassium bromate (KBrO_3_) were purchased from Sigma Aldrich (Saint Louis, MI, USA). DMEM (with high glucose, without glutamine, and phenol red) and DMSO were acquired from VWR Chemicals (Cleveland, OH, USA). Trypsin/EDTA, and pen/strep were obtained from Biochrom KG (Berlin, Germany). Trypan blue, crystal violet, methylene blue, azure II, glutaraldehyde solution, and hydrogen peroxide (H_2_O_2_) were obtained from Merck (Darmstadt, Germany). SyberGoldTM from Thermo Fisher Scientific (Waltham, MA, USA), the FPG enzyme from New England Biolabs (Barcelona, Spain), BrdU assay kit from Roche (Basel, Switzerland), and FITC-conjugated Annexin V, Annexin V buffer, and propidium iodide (PI) from BioLegend (San Diego, CA, USA).

The (+)-isomer of preussin was isolated from the marine sponge (*Epipolasis* sp.)-associated fungus, *Aspergillus candidus* KUFA 0062, collected from the coral reef of Similan Island National Park in Phang-Nga province of Southern Thailand). The process of isolation and purification of preussin has been described by Buttachon et al. [23].

Preussin (10 mM), Dox (20 mM), and SyberGold^TM^ (1000×) were prepared as stock solutions in DMSO, and MTT (5 mg/mL) was prepared in sterile PBS. The solutions were kept in aliquots at −20 °C and freshly diluted right before use.

### 4.2. Cell Culture

The MDA-MD-231 (HTB-26) cell line was purchased from American Tissue Culture Collection (ATCC). Cells were maintained as monolayer cell cultures in DMEM with high glucose, without glutamine, and phenol red supplemented with 10% FBS and 1% penicillin/streptomycin. The maintenance was made in T25 cm^2^ (with 5 mL of DMEM), and T75 cm^2^ (with 15 mL of DMEM) culture flasks (Orange Scientific, Braine-l’Alleud, Belgium) in an MCO 19AIC humidified incubation chamber (Sanyo, Osaka, Japan) at 37 °C and 5% CO_2_. Cells were regularly observed under an inverted phase-contrast CKX41 microscope (Olympus, Tokyo, Japan), and the medium was replaced every two to three days. When about 80–90% confluence was reached, cells were in their logarithmic phase and were harvested and subcultured. Cell suspensions with the desired cell density were obtained from the subcultures to perform different assays, either as monolayers or 3D cell cultures (MCAs).

### 4.3. Subculturing Conditions

Cells were washed twice with phosphate-buffered saline (PBS) [pH 7.2–7.4], detached with 0.25% trypsin 0.02% ethylenediaminetetraacetic acid (EDTA) (1 mL for cells cultured in T25 cm^2^ flasks or 3 mL for cells cultured in T 75 cm^2^ flasks) at 37 °C for 3–5 min. When cells became round-shaped and started to detach, the action of trypsin was stopped by adding supplemented DMEM in a proportion higher than 1:2 (*v*/*v*). After trypsinization, cells were centrifuged at 280× *g* for 5 min in a 416 centrifuge (Gyrozen, Gimpo, Republic of Korea), the supernatant was removed, and cells were resuspended again in DMEM. Cell density and viability of the obtained cell suspension were assessed through the trypan blue exclusion assay.

### 4.4. Trypan Blue Exclusion Assay and Determination of Cell Density

The trypan blue exclusion method was used to determine cell density and viability of cell suspensions. In this assay, cells are mixed with trypan blue. Viable cells show impermeability to the dye (appear bright under a microscope), and the non-viable cells that do not have cell membrane integrity are permeable to the dye (appear blue under a microscope) [72]. To perform the assay, 20 µL of cell suspension was added to an equal volume of 0.1% trypan blue (*w*/*v*) in PBS. Immediately, 10 µL were loaded into a Neubauer chamber, and the number of live and dead cells was counted. Cell density and viability were calculated according to the following equations:

Cell density (n° cells/mL) = average number of cells × 2 (dilution factor) × 10^4^ (conversion factor for Neubauer chamber), (1)

Cell viability (%) = (n° of viable cells)/n° of total cells × 100(2)

### 4.5. Multicellular Aggregates (MCAs)

#### 4.5.1. MCAs Obtention

To obtain MDA-MB-231 MCAs, 200 µL of cell suspension at 4 × 10^5^ cells/mL were seeded in 96-well ultra-low attachment plates (Corning, New York, NY, USA) and incubated at 37 °C, with 5% CO_2_ for 72 h. MCAs were then exposed to preussin at the desired concentrations according to the assay that was being performed.

#### 4.5.2. MCAs Disaggregation

Disintegrating MCAs using trypsin was necessary to obtain cell suspensions following exposures. Three MCAs were transferred to 1.5 mL tubes and carefully washed twice with PBS. To avoid cell loss, the cells were centrifuged with a Gyrozen 416 centrifuge between washes. For MCAs disaggregation, 100 µL of trypsin-EDTA was used, and then 300 µL supplemented medium was added to stop trypsinization. MCAs viability was assessed by trypan blue assay, as described above.

### 4.6. Viability Assay—MTT Assay

The MTT assay is a viability assay where MTT, a yellow tetrazolium salt, can be absorbed and then metabolized by the cell into formazan, a purple water-insoluble compound [91]. This reaction is carried out by mitochondrial or cytoplasmic enzymes such as dehydrogenases, oxidoreductases, oxidases, and peroxidases [91]. Mitochondrial enzymatic activity is constant in most viable cells. Subsequently, the conversion of MTT into formazan crystals allows the usage of MTT as a colorimetric assay with which it is possible to measure the increase or decrease in the number of viable cells. In this way, it is possible to infer a compound’s effect on cell proliferation and cytotoxicity activity by quantifying cellular metabolic activity [91].

#### 4.6.1. MTT in 2D Cell Culture

The MTT assay was used to evaluate the effects of preussin in MDA-MB-231 cells, as previously described [43]. MDA-MB-231 cells were seeded at a density of 5 × 10^4^ cells/mL, 100 µL of cell suspension per well in 96-multiwell culture plates (Orange Scientific, Belgium) and incubated at 37 °C and 5% CO_2_ for 24 h to allow cell adhesion. Cells were then exposed to preussin at concentrations of 5, 15, 25, 35, and 50 µM and incubated for 24, 48, and 72 h in the incubation chamber under the same conditions. Negative control with untreated cells (cells incubated only with cell culture medium), solvent control (cells exposed to 0.1% (*v*/*v*) DMSO), and positive control (cells exposed to Dox at 1 µM). Simultaneously, an additional blank control group was included in which exposure and control solutions were added to wells without cells. This procedure allowed us to obtain background Abs measurements. After the defined exposure time, 10 µL of MTT solution in PBS was added to each well and incubated for 3 h, protected from light in the incubation chamber. After MTT metabolization, the medium was completely removed, and the formed formazan crystals were dissolved by adding to each well 100 µL of DMSO/ethanol solution (in the proportion of 1:1) (*v*/*v*), and the plates were agitated for 15 min in a plate shaker. Absorbance (Abs) was read in the Multiskan™ GO Microplate Spectrophotometer (Thermo Fisher Scientific, Massachusetts, USA) at 570 nm. Data were analyzed first by subtracting the background of each condition (mean of the Abs of the blank of each exposure condition) from the mean Abs of the cells of each exposure condition, followed by the calculation of the percentage of cell viability in relation to the control, as described by Malhão et al. [43]:

Mean Abs (without background) = mean Abs of exposed cells − mean Abs of the blank(3)

Cell viability (%) = (mean Abs (without background) sample at 570 nm)/(mean Abs (without background) control at 570 nm) × 100(4)

The determination of IC_50_ was performed only for cells exposed to preussin for 72 h. The IC_50_ value was determined using GraphPad Prism 9.0 software (GraphPad Software, San Diego, CA, USA). The values of measured Abs should be proportional to the number of viable cells in the culture. MTT assay was performed in five independent experiments (n = 5), using triplicates per exposure condition. For a comparative purpose, the IC_50_ of the previous work [43] was computed using the data obtained from MTT assays that tested the effects of preussin in the same cell line.

#### 4.6.2. MTT in 3D Cell Culture

To evaluate the cytotoxic effects of preussin in MDA-MB-231 MCAs, a modified version of the MTT assay was implemented in the monolayer cell culture [43]. MCAs were obtained as described previously and were exposed to preussin at 25 and 35 µM for 24 h or, to preussin at 25, 35, and 50 µM (concentrations that previously showed effects on cell viability in 2D cell culture model after 72 h of exposure) or Dox at 5 µM (positive control) for 96 h. Following the defined period of exposure with preussin, 15 µL of MTT were added to each well and incubated for 4 h. After this time, MCAs were carefully transferred from the ULA-rounded bottom plate into a flat-bottomed culture plate with a 1000 µL micropipette tip, and the excess of the medium was carefully removed. Formazan crystals were solubilized by adding 150 µL of DMSO to each well and by agitating in a plate shaker for 20 min. The results were also expressed as a percentage of cell viability, already described in the 2D cell culture MTT assay. The MTT assay in MCAs was performed in five independent assays, using triplicates for each exposure condition.

### 4.7. In Vitro Scratch Assay

An in vitro scratch assay or cell wound-healing assay is used to measure cell migration in monolayer cultures. In this assay, a scratch is made in a confluent cell monolayer, and it is later observed at different time points to evaluate the scratch closure [102].

MDA-MB-231 cells were seeded at 2.5 × 10^5^ cells/mL allowing to obtain a confluent uniform monolayer and incubated at 37 °C and 5% CO_2_. After cell adhesion (24 h of incubation), cells were starved for 6 h with a serum-free medium for cell cycle synchronization [103]. Scratching the monolayer cell culture causes cell rupture that leads to the release of mitogenic stimuli, which in turn promotes cell migration. This period of cell starvation with the serum-free medium before scratching minimizes the cells’ endogenous migration [102].

After starvation, a scratch was made with one flowing single movement with a 100 µL micropipette tip in a vertical position and guided by a ruler to ensure that the scratch was a straight line, with a uniform width, and that the scratches of different exposure conditions had similar dimensions to minimize variability. After scratching, cell debris and suspended cells were immediately removed by washing twice with PBS so the motility-released stimuli could be quickly [102]. Cells were then exposed for 72 h to preussin at 35 and 50 µM, prepared in serum-reduced cell culture medium (DMEM), with 1% FBS (*v*/*v*) to reduce proliferation stimuli, and with 1% penicillin/streptomycin (*v*/*v*) [104]. As a negative control, cells were exposed only to the serum-reduced cell culture medium, as solvent control cells were exposed to 0.1% DMSO (*v*/*v*) in the serum-reduced cell culture medium, and internal control cells were exposed to DMEM with 10% FBS. After getting the first results, the preussin concentrations were diminished to 5, 15, and 25 µM.

Two fields of each scratch were photographed using the inverted phase-contrast microscope CKX41 microscope with an objective lens of 2× amplification and coupled with an M5DC-CYL camera (Pixellink, New York, NY, USA) at time points of 0 h, 24 h, 48 h, and 72 h. This assay was performed in 5 independent experiments with 3 replicates per condition. MDA-MB-231 cell migration was assessed, with free Fiji software (version 1.53c, National Institute of Health, Maryland, USA), at different time exposures. The acquired images were initially converted into binary images. Then, a region of interest of the scratch was selected by cropping the image laterally, by choosing the central 2/3 of the image (assessed by the Grid function of the Fiji software) and at the front of migration (horizontal line at a point with high cell density). The cropping limits were obtained by drawing a rectangle, and the area of interest was obtained by cropping the image through the selected limits of the rectangle. The area of the region of interest of the scratch was obtained with the function of “Area fraction”, which allowed the exclusion of the cells present in the middle of the scratch. The percentage of migration was evaluated as described elsewhere [105]:

Migration (%) = wound area at 0 h − wound area at 24,48, or 72 h/wound area at 0 h × 100(5)

There were 5 independent assays with triplicates per exposure condition.

### 4.8. Proliferation Assay—5′-bromo-2′-deoxyuridine (BrdU) Assay

To evaluate the effects of preussin on cell proliferation, BrdU assay was performed with the Cell Proliferation ELISA, BrdU (colorimetric) kit (Roche, Switzerland), both in monolayer and MCAs cell cultures as described by the manufacturer with some modifications, mainly for 3D cell culture, as described by Malhão et al. [43].

The BrdU assay allows for the indirect detection of cell proliferation by measuring the incorporation of BrdU into the newly synthesized DNA. BrdU is a pyrimidine analog that, in proliferating cells during cell division, will be incorporated into the DNA in substitution for thymidine. The incorporated BrdU can later be detected through ELISA techniques using anti-BrdU antibodies. This way, the number of proliferating cells in the sample should be proportional to the quantified incorporated BrdU [106].

#### 4.8.1. BrdU Assay in 2D Cell Culture

To perform the BrdU assay in MDA-MB-231 cells in 2D cell culture, 100 µL of cell suspension at a density of 5 × 10^4^ cells/mL were seeded in 96-multiwell culture plates. These were then incubated at 37 °C and 5% CO_2_ for 24 h for cell adhesion. After adhesion, the cells were exposed to 25 or 35 µM of preussin and incubated at standard conditions for 72 h. Negative control, solvent control, and positive control groups were also added. To obtain background Abs measurements, an additional blank control group in which the solutions of exposure and control groups were added to wells without cells.

After exposure, DNA was labeled by the incorporation of BrdU, adding 10 µL of BrdU labeling reagent to each well (final concentration of 10 µM) and incubating cells for 2 h at 37 °C and 5% CO_2_. After removing the labeling medium, the cells were dried for 1 h at 60 °C and stored at 4 °C overnight. The following day, the cell DNA was denatured with 200 µL of FixDenat reagent for 30 min at room temperature. The denaturing reagent was removed, and 100 µL/well of working solution of the monoclonal antibody mouse anti-BrdU conjugated with peroxidase (1:100 dilution) was added to each well and incubated at room temperature for 90 min, for detection of BrdU incorporation into cellular DNA. Furthermore, the wells were washed 3 times with 200 µL/well of washing solution (1x PBS) and incubated with tetramethylbenzidine substrate solution at room temperature for 30 min, for later photometric detection. Finally, the Abs was measured at 370 and 492 nm with the microplate reader Multiskan™ GO Microplate Spectrophotometer. The developed color, and consequently the measured Abs should directly correlate to the amount of synthesized DNA and, in this way, to the number of proliferating cells. The percentage of cell proliferation was calculated, first by obtaining the difference between the measured Abs at 370 nm and the measured Abs at 492 nm, from each well, followed by subtraction of the mean Abs of the blank groups to the mean Abs of the exposed cells. Finally, cell proliferation was calculated in relation to the control group through the following equation:

Cell proliferation (%) = Abs of sample/Abs of control × 100(6)

#### 4.8.2. BrdU Assay in 3D Cell Culture

The BrdU assay in MCAs was performed with modifications to the protocol described for monolayer cell culture. The MCAs were obtained and later exposed to preussin at 25 or 35 µM and incubated at 37 °C and 5% CO_2_ for 96 h. Negative control, solvent control, and positive control groups were also added and incubated in the same conditions. A blank control group was added, in which the solutions of exposure and control groups were added to wells without cells to obtain background Abs measurements. After exposure, 100 µL out of a total of 200 µL of medium were removed from each well (to have a final volume of 100 µL of the medium in each well), and µL of BrdU labeling reagent was added to a final concentration of 10 µM. The MCAs were incubated for 4 h at 37 °C and 5% CO_2_, for DNA incorporation of pyrimidine. The MCAs were then transferred to a flat-bottom 96 well plate, carefully removing all the exceeding culture medium. The following steps and the calculation of MCAs proliferation followed the described method for the monolayer cell culture.

### 4.9. Comet Assay

Before performing the comet assay, the test conditions were evaluated for cell viability with the MTT assay, at 24 h of exposure, to confirm whether the cell viability was higher than 70%. The genotoxicity effects of preussin were evaluated at 2 h and 24 h of exposure by the alkaline version and FPG enzymatic versions of the comet assay as described previously [86] both in monolayer and MCAs.

#### 4.9.1. Alkaline Version of the Comet Assay

The alkaline version of the comet assay or alkaline single-cell gel electrophoresis (SCGE) is used to evaluate DNA damage and the genotoxic effects of compounds in single cells. This assay detects strand breaks (double and single-strand breaks) and alkali-labile sites [107].

In a monolayer cell culture, 1 × 10^5^ cells/mL were seeded in a volume of 500 µL, in 24 well plates. For adhesion, these cells were incubated for 24 h at 37 °C and 5% CO_2_. MDA-MB-231 cells cultured in monolayer and MCAs were exposed to preussin at 25 and 35 µM, for 2 h and 24 h at 37 °C and 5% CO_2_. Simultaneously, negative control (cells treated with medium) and solvent control (cells treated with 0.1% DMSO) groups were performed. The positive control (cells treated with H_2_O_2_ for alkaline) was also included, as described below.

After exposure, cells were washed 2 times with PBS, trypsinized, and immediately transferred into an ice bath to avoid cellular repair [107]. The cell density and viability of each condition, both in monolayer and MCAs, were evaluated with the trypan blue exclusion assay using an automated cell counter Countess^TM^ (Invitrogen, MA, USA). Cells were collected in 1.5 mL tubes, centrifuged at 1500g for 10s in a MicroStar12 centrifuge (VWR, Pennsylvania, USA), and immediately transferred to ice protected from light. After supernatant removal, approximately 5 × 10^3^ cells per gel were gently resuspended in 70 µL of pre-heated LMP to 37 °C (Note: LMP agarose was kept in 2 mL aliquots at 4 °C, and each aliquot was heated only once, to avoid alteration in LMP agarose concentration caused by evaporation). This cell suspension was then pipetted into 1% (*w*/*v*) NMP agarose-coated microscope slides (prepared by dipping conventional microscope slides in liquid NMP agarose at 1% (*w*/*v*), let dry for 24 h at room temperature, and stored protected from dust) and immediately covered with a 22 × 22 mm coverslip. The slides were transferred to a cold horizontal surface to obtain the gel and kept at 4 °C for 10 min for complete gel solidification. The coverslips were carefully removed by sliding them through the gel. Per experimental condition, 3 slides (with 2 gels each) were performed (one to be kept in a lysis solution, the other in buffer F, and the last to incubate with FPG). To be used as a positive control for the standard alkaline version of SCGE, two additional slides from the negative control conditions (2D or 3D cell culture) were also collected. At this stage, to obtain the positive control of the standard version of the alkaline SCGE, 2 gels of the same slide, with cells from the control conditions (from 2D or 3D cell culture), were either exposed to cold 50 µM of H_2_O_2_ in PBS or PBS, for 5 min on ice protected from light and later washed with cold PBS.

For cell lysis and, consequently, DNA release, all the slides were treated with a lysis solution (2.5 M NaCl, 100 mM Na2EDTA, 10 mM Tris Base, at pH 10, with 1% (*v*/*v*) Triton X-100 added right before use) for 1 h at 4 °C in the dark. Slides from the positive control and exposure groups were incubated separately in the lysis solution to avoid DNA damage caused by H_2_O_2_ residues. After lysis, all the slides were washed with distilled water and in cold electrophoresis buffer (0.3 M NaOH and 1 mM Na2EDTA, pH > 13). For DNA unwinding, the slides were transferred into the electrophoresis chamber and incubated in the electrophoresis buffer for 40 min at 4 °C protected from light. Later, the electrophoresis of the gel-embedded bodies, nucleoids, was taken at 21 V (1 V/cm) for 20 min at 4 °C, protected from light. The slides were washed with distilled water three times for 5 min each and then dehydrated in absolute ethanol three times for 5 min each. After dehydration, they were left to dry at room temperature.

Right before analysis, the slides were stained for 30 min of SyberGoldTM staining solution, at a dilution of 10,000× in Tris-EDTA (TE) Buffer (10 mM Tris-HCl and 1 mM EDTA, pH 7.5), under slight agitation and protected from light. Slides were washed with deionized and left to dry at room temperature, also in the dark, and analyzed with a Motic BA410 ELITE microscope, attached to an epi-fluorescence illuminator Motic MXH-100 power supply for HG 100W, with 100× magnification (Filter Set: Exciter D480/30x, Emitter D535/40nm, Dichroic 505DCLP) (Motic, Kowloon, Hong Kong, China). For each condition, 100 non-overlapping, randomly selected comets were evaluated from duplicated gels (50 from each gel), avoiding the comets from the edge of the gels. For comet analysis and image capture, Comet Assay IVTM software (Instem PLC, Staffordshire, UK) was used, and the cell DNA damage was measured through the percentage of DNA in the comet tail (%tDNA). The results are expressed as %tDNA of five independent assays, each performed on different days.

#### 4.9.2. Detection of FPG-Sensitive Sites

Cells quickly repair single-strand breaks. Therefore, they may represent less relevant DNA damage than other types of DNA damage that can be mutagenic and have higher effects on viability and genetic stability. For this reason, we evaluated the effects of preussin on DNA oxidative damage by detecting FPG-sensitive sites. The FPG enzyme can remove oxidized purines (such as 8-oxo-7,8-dihydroguanine or formamidopyrimidines) and adducts of ring-opened N7 guanine formed by alkylating agents [84].

For this assay, 4.5 mM of KBrO_3_ in supplemented DMEM (100 µL in monolayer cell culture and 200 µL in MCA’s), at standard incubation conditions for 1 h, was selected as a positive control for the enzymatic version of the comet assay as described previously [108]. The positive controls were optimized for the MDA-MB-231 cell line, cultured at monolayer and MCAs cell culture conditions. The gels were obtained as described above in the standard version of the comet assay, and the slides were washed after lysis three times for 5 min at 4 °C with cold buffer F (40 mM HEPES, 0.1 M KCl, 0.5 mM EDTA, 0,2% (*w*/*v*) BSA). After removing the excess buffer F, the gels were incubated with 50 µL of either buffer F or FPG enzyme at a dilution of 1:10,000 in buffer F (optimized for this cell line). The gels were covered with a 22 × 22 mm coverslip and incubated in a moist box at 37 °C for 30 min, protected from light. The alkaline treatment, electrophoresis, dehydration, staining, and %tDNA quantification, were carried out as described above. For the evaluation of FPG-sensitive sites, the mean of the %tDNA from buffer F incubated slides was subtracted from the obtained mean of the %tDNA of the slides incubated with the FPG enzyme. Five independent assays were performed, each on a different day.

### 4.10. Annexin V—PI Assay

The annexin V—PI assay detects apoptotic membrane changes, distinguishing early apoptosis from late apoptosis/necrosis [51]. Annexin V is a protein capable of sensitively binding, in a calcium-dependent manner, the phosphatidylserine residues of phospholipids, which are normally found in the innermost layer of the cell membrane but that during early apoptosis is translocated to the outer phospholipid layer. If a DNA labeling compound is simultaneously used, such as PI, it is possible to distinguish early apoptotic from late apoptotic and necrotic cells where membrane disruption allows the DNA to be stained by PI. In this way, viable cells will appear unstained, early apoptotic cells will stain only with the labeled Annexin V, and the late apoptotic and necrotic cells will appear both stained by the labeled Annexin V and by PI [51,90].

To analyze if preussin could induce cell death and whether it would be due to apoptosis or necrosis, Annexin V—PI staining evaluation was performed by flow cytometry.

For monolayer cell culture, 2 × 10^5^ cells/well were seeded in 6-well plates at a density of 1 × 10^5^ cells/mL and left to adhere for 24 h and exposed to preussin at 25 and 35 µM for 72 h. After exposure, the supernatant was collected, and the remaining adherent cells were collected by washing two times with PBS followed by incubation with 500 µL of trypsin-EDTA for 3 min. Tripsinization was stopped by adding 1 L of supplemented cell culture medium, and the cells were added to the cells collected from the supernatant.

In 3D cell culture, three MCAs per triplicate were obtained and were exposed for 96 h to the selected conditions of preussin at 25 and 35 µM. Thereafter, the supernatant and the MCAs were collected into 1.5 mL tubes, and the cell disaggregation proceeded with trypsin-EDTA as described previously. Simultaneously, negative control, solvent control, and positive control (1 µM of Dox in 2D culture or 5 µM in MCAs) groups were added. To be used subsequently for the correction of the fluorescence data, additional MCAs and monolayer cell cultures of the negative control were maintained.

After cell collection, the monolayer and MCAs-derived cell suspensions were centrifuged at 300 g, the supernatant removed, and the cells resuspended in 50 µL of Annexin V buffer. Then, 50 µL of the cell suspension was transferred into flow cytometry tubes, and the cell suspensions were then incubated with 0.5 µL of FITC-conjugated Annexin V for 15 min at room temperature, protected from light. Right before analysis, 0.5 µL of PI at 0.25 mg/mL was added to each cell suspension. Cell analysis was performed using a FACSCanto II flow cytometer (BD Biosciences, Franklin Lakes, NJ, USA) and the BD FACSDiva™ Software. Flow cytometry controls were obtained (Annexin V, PI, and unstained controls) from the negative control cells (both from MCAs and monolayer cultures) for later correction of fluorescence readings. The results were analyzed using the FlowJo™ v10 Software (BD Biosciences). Before analysis, the data samples were corrected for fluorescence spillover emissions, a process called compensation, with the usage of single stained controls (stained only with Annexin V or PI). After compensation, the samples were gated, first to exclude debris and, secondly, for the selection of single cells (with the exclusion of aggregated cells) (Figure S9). Finally, four-quadrant gates were defined based on the single-stained controls. In this way, three cell populations were defined: viable cells (Annexin V−/PI), dead cells (Annexin V+/PI+ or Annexin V−/PI+), and apoptotic cells (Annexin V+/PI−). The assay was performed in 5 independent replicates (each on a different day) with triplicates for each replicate.

### 4.11. Morphological Analysis

#### 4.11.1. Phase-Contrast Microscopy and Stereomicroscopy

For every executed assay, either in a monolayer or 3D cell culture model, a daily analysis of the morphological alterations caused by the exposure to preussin was performed. Whenever the defined time point was reached and before proceeding with the assay, representative images of the structural changes in each condition were acquired (for every assay and cell culture model). For the monolayer cell culture, the cells were photographed with a CKX41 (Olympus, Tokyo, Japan) inverted phase-contrast microscope using an M5DC-CYL camera (Pixelink, ON, Canada). As for 3D cell culture, the MCAs were photographed with an SZX10 stereomicroscope using the darkfield filter and a DP21 camera (Olympus, Tokyo, Japan) and the areas were measured with the AnaSP freeware [109].

#### 4.11.2. Transmission Electron Microscopy (TEM)

Cells from monolayer cultures and MCAs were collected and processed for TEM to investigate ultrastructural alterations caused by preussin. For monolayer cell culture, 2 × 10^5^ cells/well were seeded in 6-well plates at a density of 1 × 10^5^ cells/mL, for 24 h at 37 °C and 5% CO_2_. The MCAs and the cells in monolayer cell culture were exposed to preussin at 5, 15, 25, and 35 µM, either for 72 h (monolayer cell culture) or 96 h (MCAs), at 37 °C and 5% CO_2_. Simultaneously, negative control, solvent control, and positive control (1 µM of Dox in 2D culture or 5 µM in MCAs) groups were added. After the defined time exposures, monolayer cell culture cells were harvested through trypsinization by washing two times with PBS and adding 500 µL of trypsin-EDTA. After 3 min, the effect of the enzyme was stopped by adding 1 mL of supplemented cell culture medium, and the cells were collected into 1.5 microtubes and centrifuged for 5 min at 280 g. The supernatant was removed, and the cells were immediately fixed for 2 h at 4 °C, with freshly prepared 2.5% glutaraldehyde in sodium cacodylate-HCl buffer (0.1 M, pH 7.2). In MCAs, after exposure, 150 µL of cell culture medium was removed, and the same volume of fixative was added for 15 min. The MCAs were then collected into 1.5 mL tubes with a 1000 µL pipette whose tip was cut off to enlarge its diameter and avoid MCAs destruction. The exceeding medium was removed, and the MCAs were fixed as described for the monolayer cultures.

After fixation, cells were washed 2 times with the same 0.1 M cacodylate buffer (10 min each) and post-fixed in 1% osmium tetroxide in the same buffer for 2 h at 4 °C. Cells were then washed 2 times with 0.1 M cacodylate buffer (10 min each) and dehydrated in 50% ethanol, 70%, ethanol, 96% ethanol, two times in absolute ethanol, and two times in propylene oxide, for 30 min in each step. Cells were then gradually embedded in epoxy resin through the usage of a gradient of concentrations of the epoxy with propylene oxide (respectively, in the proportions of 1:3; 1:1, and 3:1), and a final solution of only epoxy resin, for 1h in each step, plus 10 min at 60 °C for evaporation of propylene oxide residues. At last, the monolayer cells and the MCAs were placed in electron microscopy rubber embedding molds with freshly prepared epoxy resin and incubated for 48 h at 60 °C, for resin polymerization. After polymerization, the hardened blocks were manually trimmed with a razor blade and later sectioned with an EM UC7 ultramicrotome (Leica, Wetzlar, Germany) with diamond knives (Diatome, Nidau, Switzerland). First, 1 µm semithin sections were collected onto adhesive glass microscope slides (VWR, PA, USA) and stained with a 1:1 mixture of 1% methylene blue and 1% azure II. After washing with distilled water, drying at 60 °C, and mounting with the synthetic mounting medium Bio Mount HM (Bio-Optica, Milano, Italy), the slides were observed under a BX50 microscope (Olympus, Tokyo, Japan) and photographed with an EP50 Microscope Digital Camera (Olympus, Tokyo, Japan) camera. For ultrastructural analysis, 90 nm thick ultrathin sections were obtained [110]. The grids were later observed under a JEOL 100CXII transmission electron microscope (JEOL, Tokyo, Japan), operated at 60 kV. Representative photographs of the ultrastructural changes were captured using an Orius SC1000 CCD digital camera (Gatan, CA, USA). The assay was performed using independent duplicates.

For semiquantitative analysis of lipid and MVDB content in the different exposure groups of the 2D and 3D cultures, 130 images were graded on their content, following the criteria described in Tables S1 and S2 in the Supplementary Materials.

### 4.12. Statistical Analysis

Statistical analysis was performed using GraphPad Prism 9.0 software. The results are presented as mean ± standard deviation (SD) from five independent assays. The differences between groups were first evaluated using One-Way ANOVA. When the ANOVA displayed significant (*p* < 0.05), the test was followed by post-hoc Holm–Šídák multiple comparisons (between the negative control and the exposed groups). The ANOVA assumptions of normality and homogeneity of variance were confirmed by the Shapiro–Wilk and Levene tests, respectively, using the PAST 4.09 software [111]. Whenever the homogeneity of variances was not observed, the differences were initially assessed by the Welch ANOVA test. In the case of significance (*p* < 0.05), the post-hoc Dunnett’s T3 multiple comparisons test was applied. The statistical significances of the results are indicated with one, two, three, or four asterisks (*), corresponding, respectively, to p-values inferior to 0.05, 0.01, 0.001, and 0.0001. For the semiquantitative analysis of MVB and lipid droplets content, the obtained data were converted to ranks using the PAST 4.09 software [111], and differences between the models of culture and exposure conditions were assessed by two-way ANOVA followed by the post-hoc Tukey multiple comparisons test.

## Data Availability

The data supporting this study’s findings are available from the corresponding author upon reasonable request.

## Supplementary Materials

The following supporting information can be downloaded at: https://www.mdpi.com/article/10.3390/md21030166/s1, Figure S1: Dose response curve of cell viability in the MDA-MB-231 cells exposed to preussin for 72 h, assessed by the MTT assay; Figure S2: Effects of preussin on cell viability in MDA-MB-231 MCAs, assessed by the MTT assay after 24 h; Figure S3: Representative effects of preussin at 5, 15, 25 and 35 µM on cell migration assessed by the in vitro scratch assay; Figure S4: Effects of preussin at 25 and 35 µM on cell viability after 2 h or 24 h of exposure, assessed through the trypan blue exclusion assay; Figure S5: Representative effects of preussin on the fresh morphology of cells cultured in monolayer; Figure S6: Representative effects of preussin on MCAs morphology in darkfield stereomicroscopy; Figure S7: Effects of preussin at 25, 35 and 50 µΜ on MDA-MB-231 MCAs area after 96 h of exposure; Figure S8: Semiquantitative analysis of the effects of preussin and the cell culture model on MVDB (a) and lipid droplet content (b); Figure S9: Representative images of flow cytometry gating strategy used in the annexin V-FITC/PI double staining assay; Table S1: Semiquantitative ultrastructural grading analysis of the cell content in multivesicular dense bodies (MVDB); Table S2: Semiquantitative ultrastructural grading analysis of the cell content in lipid droplets.
