# Supplementary material for: Effects and Mechanisms of Action of Preussin, a Marine Fungal Metabolite, against the Triple-Negative Breast Cancer Cell Line, MDA-MB-231, in 2D and 3D Cultures
Source: Mar Drugs. 2023 Mar 4;21(3):166. doi: 10.3390/md21030166 (PMC10053333; doi:10.3390/md21030166)
Supplement: Supplementary file 1 [file marinedrugs-21-00166-s001.zip › marinedrugs-2158811-supplementary.pdf]

marine drugs

MDPI

Supplementary Materials

Effects and Mechanisms of Action of Marine-Derived Preussin against the TNBC Cell Line MDA-MB-231 in 2D and 3D Cultures

Rosária Seabra [1,2], Fernanda Malhão [1,2], Alexandra Correia [1,3], Carla Costa [4,5], Anake Kijjoa [1,2,*] and Eduardo Rocha [1,2,*]

[1] ICBAS—Instituto de Ciências Biomédicas de Abel Salazar, Universidade do Porto, Rua de Jorge Viterbo Ferreira, 228, 4050-313 Porto, Portugal
[2] CIIMAR—Centro Interdisciplinar de Investigação Marinha e Ambiental, Universidade do Porto, Terminal de Cruzeiros do Porto de Leixões, Av. General Norton de Matos s/n, 4450-208 Ma-tosinhos, Portugal
[3] I3S—Instituto de Investigação e Inovação em Saúde, Universidade do Porto, Rua Alfredo Allen, 4200-135 Porto, Portugal
[4] Department of Environmental Health, INSA—National Institute of Health Dr. Ricardo Jorge, R. de Alexandre Herculano 321, 4000-053 Porto, Portugal
[5] ITR—Laboratório para a Investigação Integrativa e Translacional em Saúde Populacional, EPIUnit—Instituto de Saúde Pública da Universidade do Porto, Rua das Taipas 135, 4050-600 Porto, Portugal
* Correspondence: ankijjoa@icbas.up.pt (A.K.); erocha@icbas.up.pt (E.R.); Tel.: +351-22-042-8331 (A.K.); +351-22-042-8245 (E.R.)

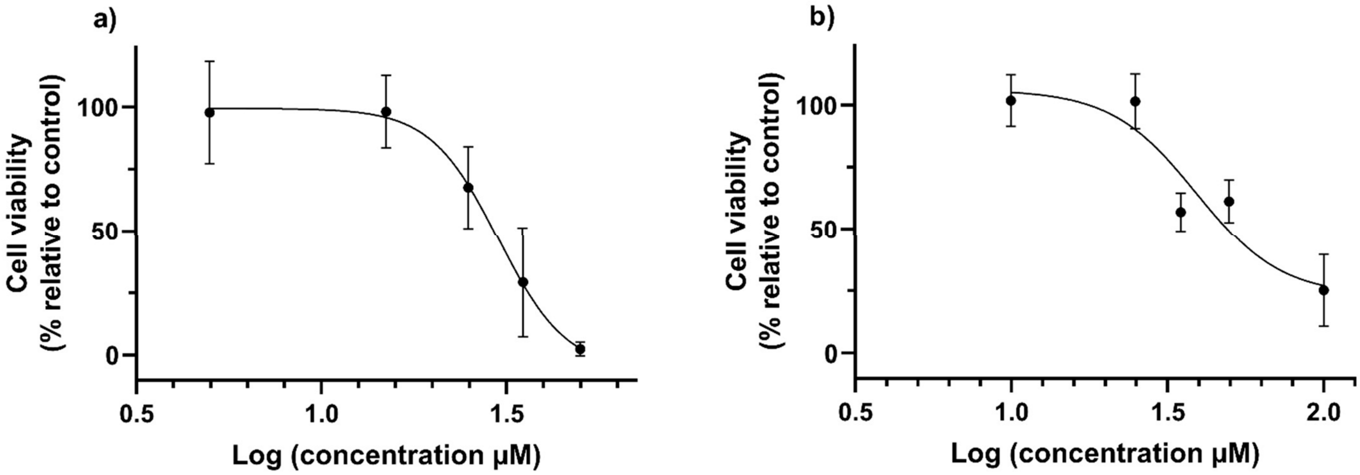

Figure S1. Dose-response curve of cell viability in the MDA-MB-231 cells exposed to preussin for 72 h, assessed by the MTT assay; in this study (a) and within the context of trials made in the previous study [43] (b). The results are expressed as the percentage of cell viability relative to a negative control (cells exposed to cell culture medium) and are expressed as mean ± SD; n = 5 (a); n = 4 (b).

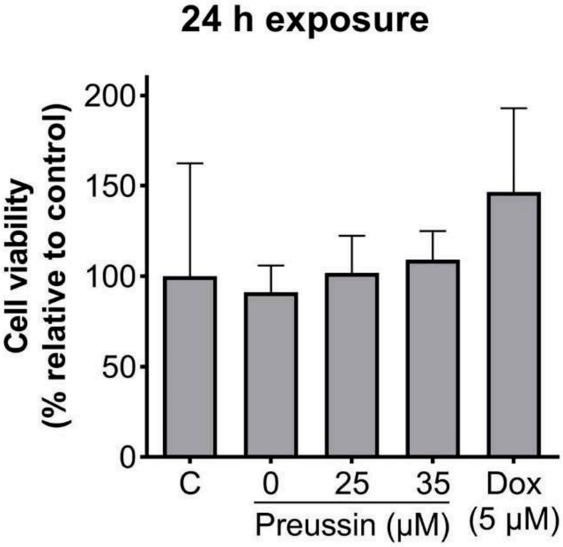

Figure S2. Effects of preussin on cell viability in MDA-MB-231 cells cultured as MCAs, assessed by the MTT assay after 24 h. Negative control (C) (cells in cell culture medium), solvent control (0 µM of preussin and 0.1% of DMSO), and positive control (cells exposed to 5 µM of Dox) groups were included. The results are expressed as the percentage of cell viability, relative to the negative control, and are expressed as mean ± SD (n = 5).

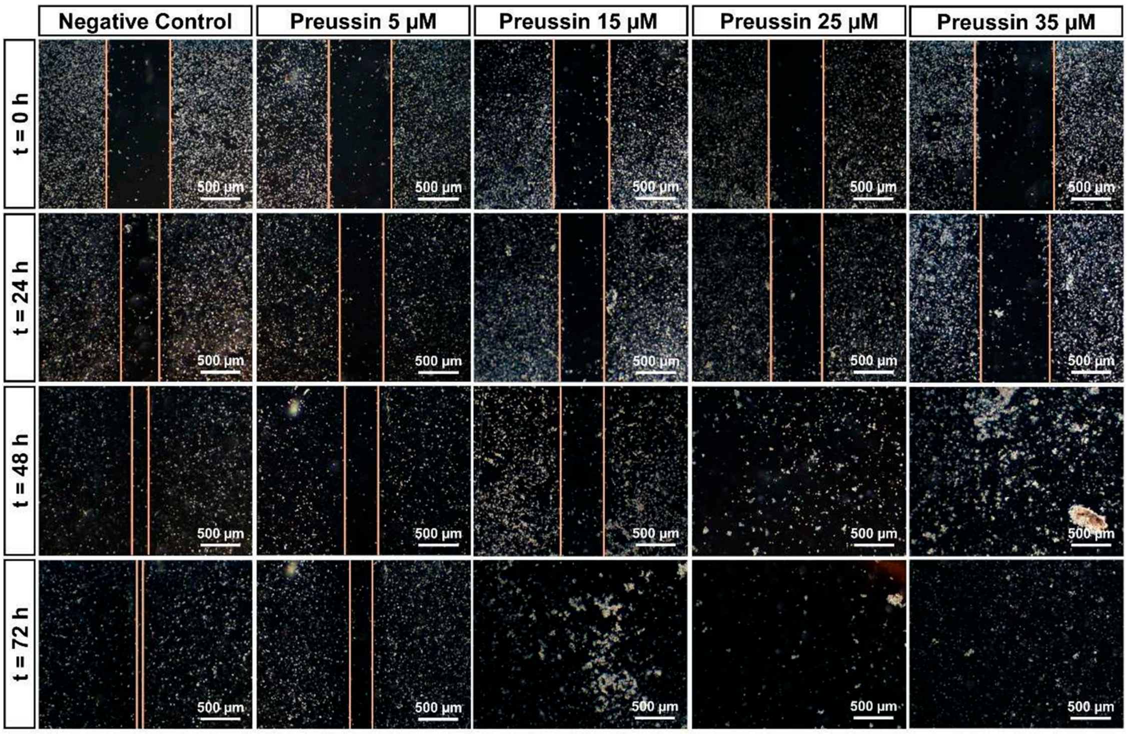

Figure S3. Representative effects of preussin at 5, 15, 25 and 35 µM on cell migration assessed by the scratch assay. Cells were seeded in a monolayer at a high density, serum-starved for 6 h, scratched, and exposed to the compounds in the culture medium supplemented with 1% FBS. The scratches were photographed in an inverted phase-contrast microscope at 0 h, 24 h, 48 h, and 72 h of exposure. The cells' front of migration is marked with orange lines. As solvent control, cells were exposed to the culture medium with 1% FBS and 0.1% of (v/v) of DMSO.

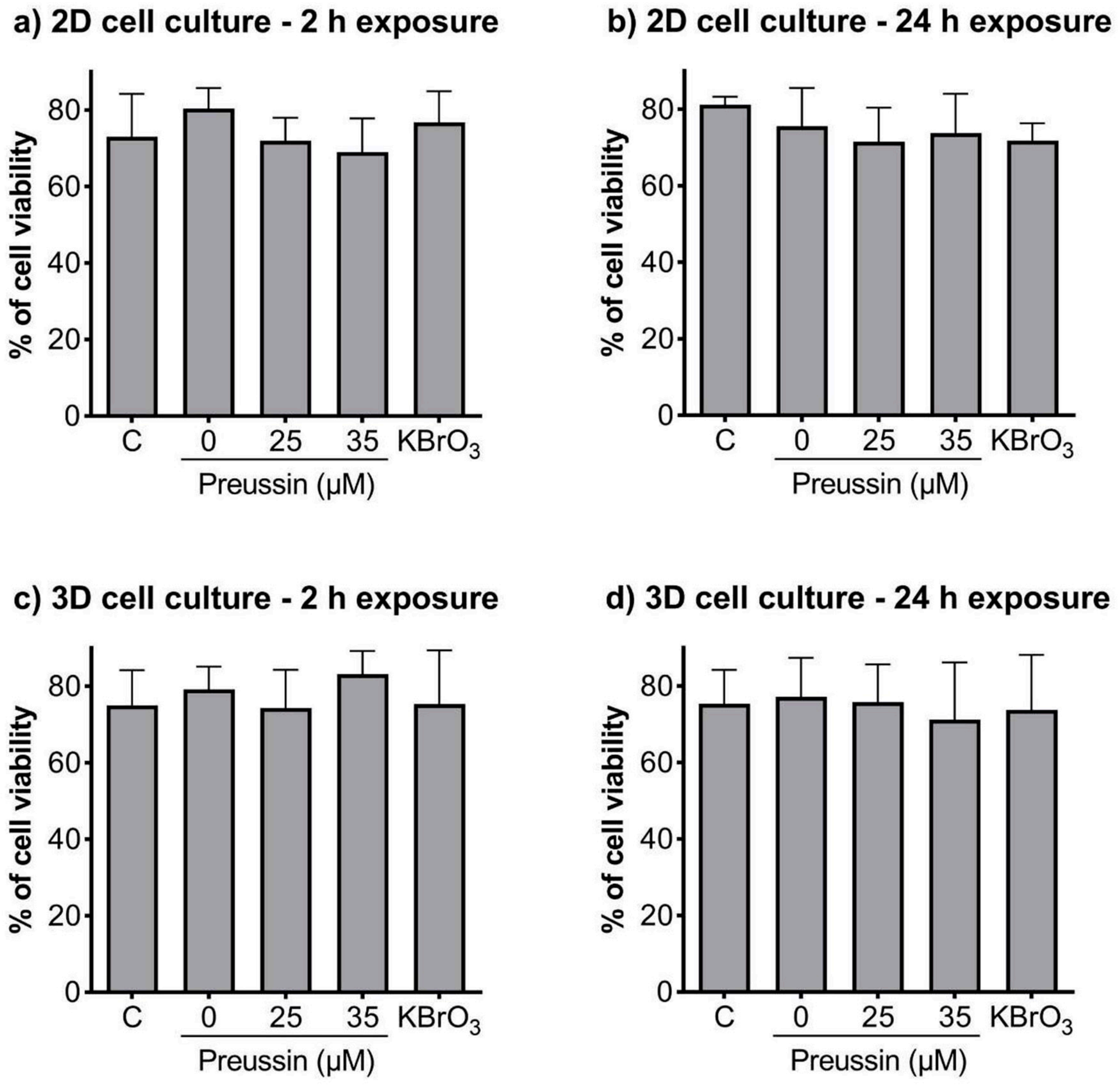

Figure S4. Effects of preussin at 25 and 35 μM on cell viability of MDA-MB-231 cells cultured in 2D (**a** and **b**) or 3D (**c** and **d**), after 2 h (**a** and **c**) or 24 h (**b** and **d**) of exposure. Cell viability was assessed through the trypan blue exclusion assay using an automated cell counter Countess™. Negative control (C) with cells in culture medium, solvent control (0 μM of preussin and 0.1% of DMSO) of cells exposed to 0.1% (v/v) of DMSO, and positive control for the enzymatic version of comet assay with cells exposed to 4.5 mM of $KBrO_3$, groups were included. The results are expressed as mean ± SD of the percentage of cell viability (n = 5).

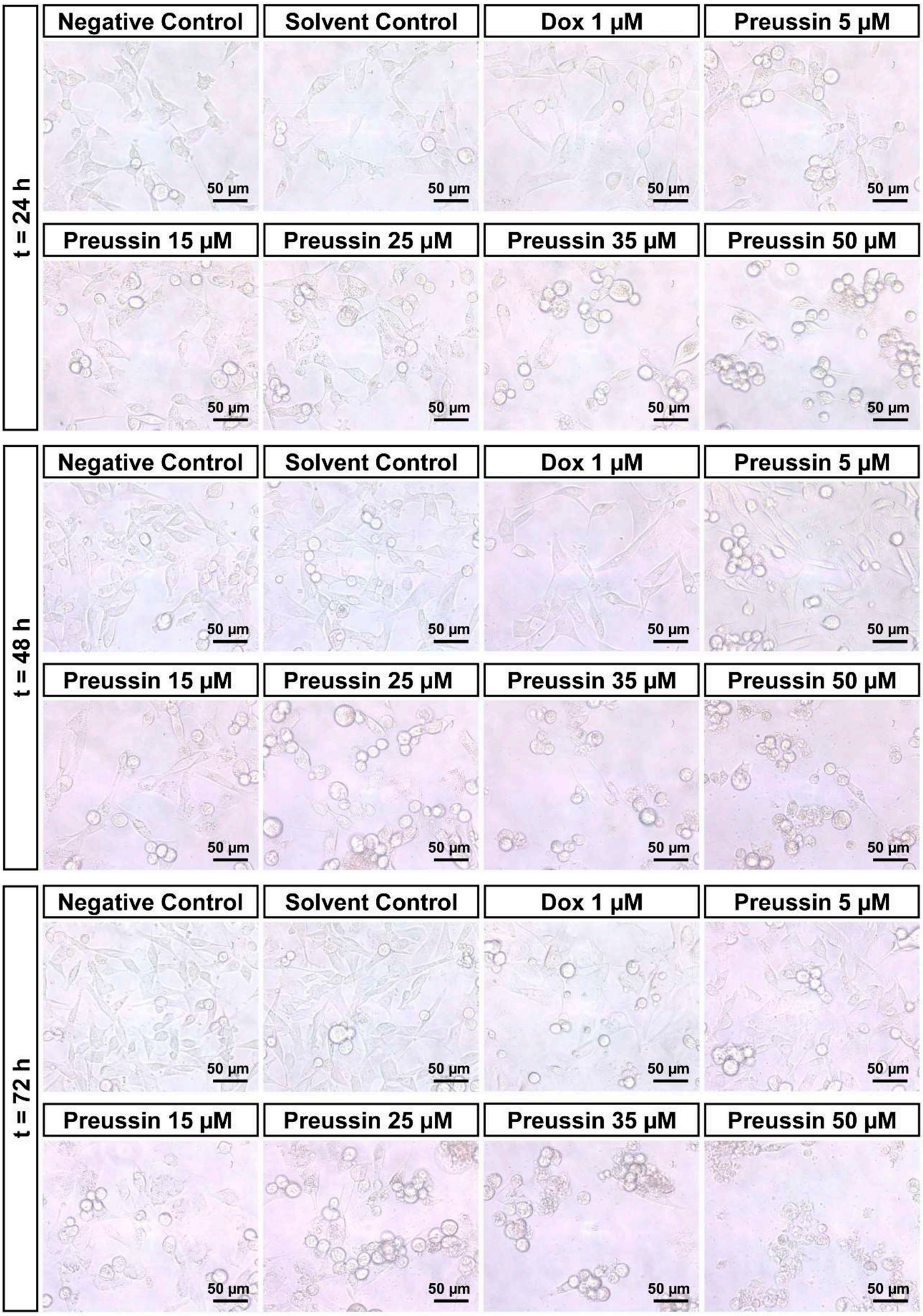

Figure S5. Representative effects of preussin on the morphology of cells cultured in monolayer. The fields were photographed in an inverted phase-contrast microscope. Cells were exposed to preussin at 5, 15, 25, 35, or 50 µM, during 24, 48, and 72 h. Cells exposed only to the cell culture medium, to 0.1% of DMSO or 1 µM of Dox, were included as negative, solvent, and positive control groups, respectively.

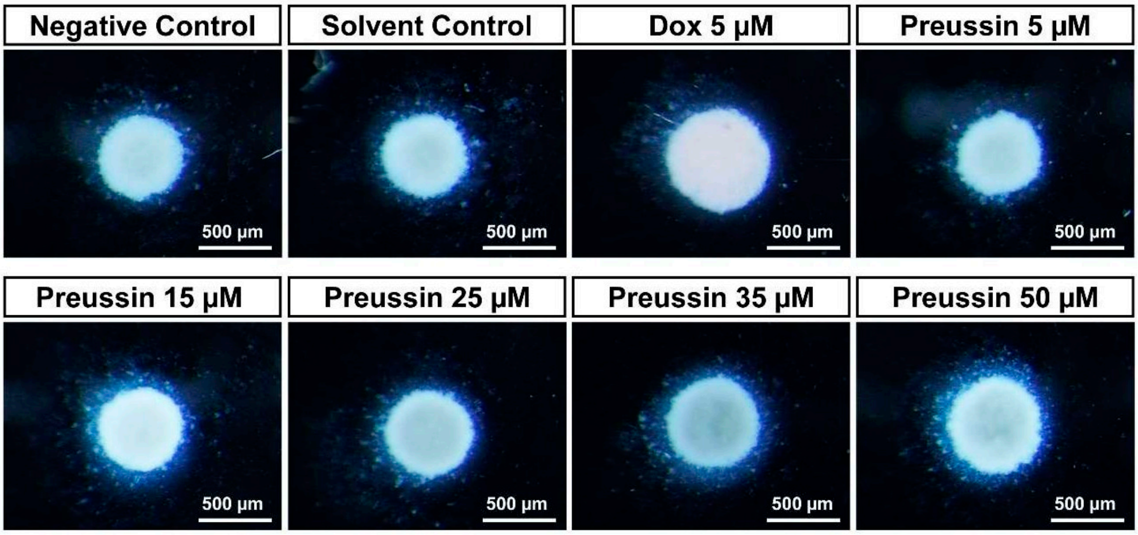

Figure S6. Representative effects of preussin on MCAs morphology in dark field stereomicroscopy. MCAs were exposed to preussin at 5, 15, 25, 35, or 50 μM, for 96 h. Cells exposed only to the cell culture medium, to 0.1% of DMSO or 5 μM of Dox, were included as negative, solvent, and positive control groups, respectively.

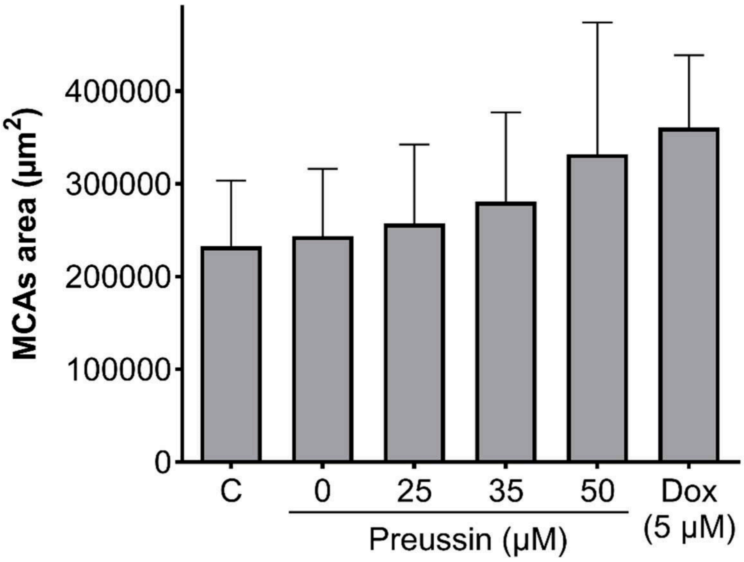

Figure S7. Effects of preussin at 25, 35 and 50 μM on MDA-MB-231 MCAs area after 96 h of exposure, measured trough the AnaSP software. Negative control (C) with cells in culture medium, solvent control (0 μM of preussin and 0.1% of DMSO) of cells exposed to 0.1% (v/v) of DMSO, and positive control of Dox (5 μM of preussin and 0.1% of DMSO). The results are expressed as mean + SD of the area in $μm^2$ (n = 5).

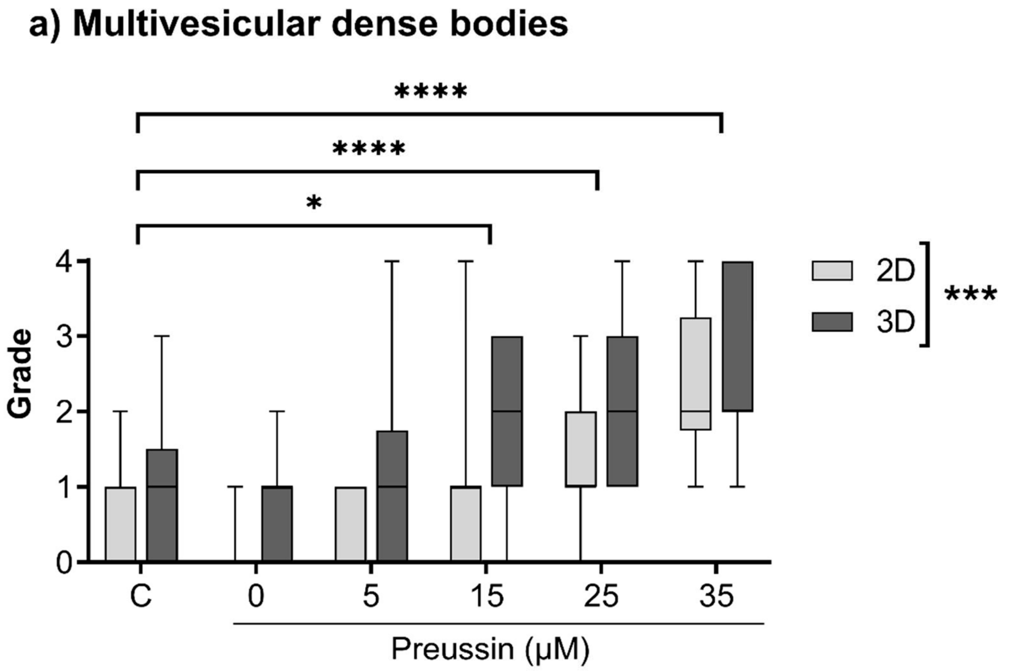

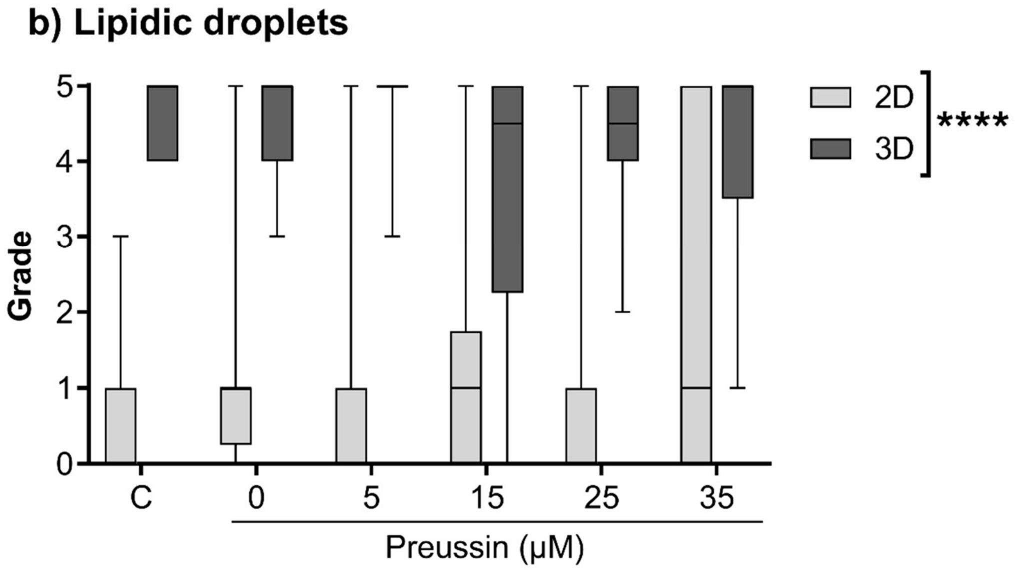

Figure S8. Effects of preussin at 5, 15, 25 and 35 μM in MDA-MB-231 cells cultured in monolayer and MCAs, on MVDB (**a**) and lipid droplet content (**b**), assessed trough semiquantitative analysis of graded levels of these structures. Negative control (C) with cells in culture medium and solvent control (0 μM of preussin and 0.1% of DMSO) of cells exposed to 0.1% (v/v) of DMSO. Data is expressed in boxplots with the minimum, median, maximum, and interquartile range Q1-Q3 (a total of 130 ultrastructural images were graded). Statistically significant differences were found in the grades of MVDB and lipidic droples between the model of culture (2D or 3D) and in the grade of MVDB for the exposure group (C, and preussin at 0, 5, 15, 25 and 35 μM). No interactions were found between factors. Differences were evaluated by two-way ANOVA, followed by the post-hoc Tukey's test (in the groups of exposure in the MVDB grading, by grouped by the model of culture, in comparison to the C) and are presented with asterisks (* $p<0.05$; *** $p<0.001$; **** $p<0.0001$).

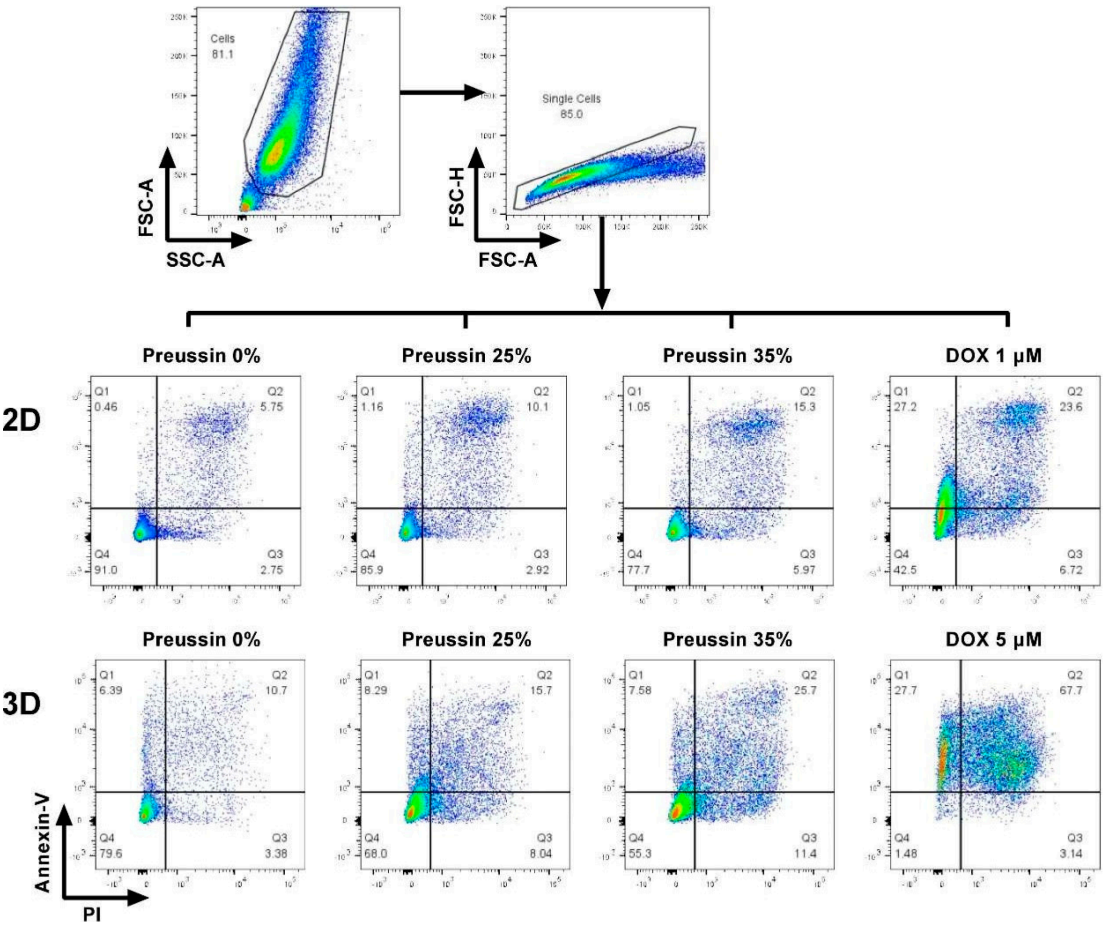

Figure S9. Representative images of flow cytometry gating strategy used to analyze annexin V/PI staining of MDA-MB-231 cells, cultured in 2D and 3D. First, the MDA-MB-231 cells were selected with the exclusion of cell debris, followed by the selection of single cells and exclusion of cell aggregates. The gated cells from each exposure group and culture model were then characterized based on the PI/Annexin V staining: Q1 + Q2 (annexin+ or -/ PI+) represented dead or dying cells, such as cells in necrosis/late apoptosis; Q3 (Annex-in+/ PI-) represented the cells in early apoptosis; Q4 (annexin-/ PI) represented the living cells. A: area; FSC: forward-scattered light; H: height; PI: propidium iodide; SCC: side-scattered light.

Table S1. Semiquantitative ultrastructural grading analysis of the cell content in multivesicular dense bodies (MVDB).

Semiquantitative grade	Qualitative grading	Area % occupied by the MVDB in the cell cytoplasm
0	Absent/ Low	0% - 5%
1	Medium	6% - 25 %
2	High	26% - 50 %
3	Very high	51% - 75%
4	Extreme	≥ 76%

Table S2. Semiquantitative ultrastructural grading analysis of the cell content in lipid droplets.

Semiquantitative grade	Qualitative grading	Number of lipid droplets per field of view with cells
0	Absent	0
1	Very low	1 - 2
2	Low	3 - 4
3	Medium	5 - 6
4	High	7 - 8
5	Very high	≥ 9
